# Suppression of sucrose synthase affects auxin signaling and leaf morphology in tomato

**DOI:** 10.1371/journal.pone.0182334

**Published:** 2017-08-07

**Authors:** Shlomo Goren, Nitsan Lugassi, Ofer Stein, Yelena Yeselson, Arthur A. Schaffer, Rakefet David-Schwartz, David Granot

**Affiliations:** Institute of Plant Sciences, Agricultural Research Organization, The Volcani Center, Bet Dagan, Israel; University of Tsukuba, JAPAN

## Abstract

Metabolic enzymes have been found to play roles in plant development. Sucrose synthase (SUS) is one of the two enzyme families involved in sucrose cleavage in plants. In tomato, six SUS genes have been found. We generated transgenic tomato plants with RNAi suppression of *SlSUS1*, *SlSUS3* and *SlSUS4* genes. Independent transgenic lines with RNAi suppression of more than one *SUS* gene exhibited morphological effects on their cotyledons and leaf structure, but there were no significant effects on their carbohydrate levels, demonstrating that SUS has a developmental function, in addition to its metabolic function. Shoot apices of the transgenic lines showed elevated expression of *JAGGED* (*JAG*) and the auxin transporter *PIN1*. In a PIN1-GFP fusion reporter/SUS-RNAi hybrid, PIN1-GFP patterns were altered in developing leaves (as compared to control plants), indicating that *SlSUS* suppression alters auxin signaling. These results suggest possible roles for SUS in the regulation of plant growth and leaf morphology, in association with the auxin-signaling pathway.

## Introduction

In plants, sugars function not only as metabolic resources and structural components, but also as regulators of various processes related to growth and development at all stages of life, from germination to senescence [1–8]. In many plants, including tomato (*Solanum lycopersicum*), carbon fixed in photosynthetic (source) tissues is either stored as starch in the chloroplast or transported to sink tissues, primarily in the form of the non-reducing disaccharide sucrose (glucose-1-(α-α)-6-fructose, Suc). Sucrose is transported through the phloem to sink tissues, where it must be cleaved before it can enter metabolic pathways.

In plants, sucrose cleavage in sink and source tissues is carried out by members of two separate enzyme families, invertases that cleave sucrose into glucose and fructose and sucrose synthases (SUS) that cleave sucrose into fructose and UDP-glucose. Both families are comprised of multiple isozymes. At least five invertases and six sucrose synthase genes have been described in tomato [9–13]. Three SUS genes, *SlSUS1*, *SlSUS3* and *SlSUS4*, were cloned prior to the publication of the tomato genome sequence [10, 13, 14] and another three SUS genes, *SlSUS5*, *SlSUS6* and *SlSUS7*, were identified in the tomato genome [12]. There is no *SlSUS2* because the sequence initially referred to as *SUS2* (GenBank acc. AJ011535) was later identified as *SlSUS1*. The existence of multiple isoforms of a metabolic enzyme leads to questions of specialization and/or redundancy of these genes. In many plants, such as Arabidopsis [15, 16] and potato (*Solanum tuberosum*) [17], different *SUS* genes are expressed in different spatial and temporal patterns. In tomato, the expression patterns of one *SUS* gene, *SlSUS1*, appear to be coordinated with stem vascular maturation [10].

The role of SUS, in general, and the roles of specific SUS isozymes have been studied using mutant or transgenic plants with altered expression of one or more isozymes. In Arabidopsis, which has six SUS genes, the exclusion of multiple genes through mutant crosses did not significantly affect plant viability or morphology [15, 16]. However, these mutants shed light on specific roles of certain isozymes. The *sus2-sus3* double-mutant displayed abnormalities in the allocation of carbon to the seeds during development, which also affected seed maturation. However, despite those differences, the mature seeds were not significantly different from control seeds [18]. Several studies have suggested that heterologous overexpression of the *SUS* genes in plants promotes the production of biomass [19–24]. These studies focused on changes in soluble sugars and biomass in transgenic plants with ectopically expressed *SUS*. *SUS* antisense studies in several species, such as potato, carrot and tomato, focused on carbohydrate levels (sugar, starch or cellulose). The tubers of potato plants with antisense silencing of *StSUS4* have reduced starch content [25]. Accordingly, overexpression of *StSUS4* in potato leads to an increase in starch levels [26]. Silencing of SUS in cotton plants leads to impaired development of seed fibers, characterized mainly by cellulose synthesis [27]; whereas overexpression of *StSUS* in cotton has the opposite effect [24]. However, these studies also showed that SUS modulation affects growth processes. For example, suppression of a *SUS* gene in carrot leads to reduced growth, correlated with SUS activity levels [28]. In tomato, antisense suppression of *SlSUS1* under a constitutive promoter leads to reduced fruit set, correlating with SUS activity, without significantly altering the carbohydrate balance in the fruit [29]. Notably, these effects were not observed when suppression was driven by a fruit-specific promoter [30], suggesting that SUS activity outside the fruit is involved in fruit-setting. Given the role of metabolic enzymes in plant development and the apparent association of SUS expression with developing tissues, in this study, we set out to further examine possible developmental roles of SUS by refining the patterns of expression of the different isoforms and studying the effects of modulation of their expression.

## Materials and methods

### Plant materials

Experiments were performed on tomato plants (*Solanum lycopersicum* cv. MP1) and transgenic constructs or hybrid crosses thereof as described below. Unless otherwise noted, unmodified MP1 plants served as a control group in all experiments. Plants were grown in a soil mixture of 70% tuff and 30% peat (Shaham-ADA, Israel) in a greenhouse under natural light conditions during two seasons: winter (15–25°C, 10 h of daylight) and summer (20–30°C, 14 h of daylight).

### Transgenic lines

For the SUS-GUS lines (proSlSUS), segments running from about 1100–1400 bp upstream of the transcription start site to the ATG start site of each of the *SlSUS* genes were cloned from genomic DNA. These segments included the large 5' UTR introns contained in the SUS genes [31] and were 2812 bp, 2952 bp and 2347 bp in length for SUS1, SUS3 and SUS4, respectively. The segments (shown in S1 File) were each subcloned into the binary vector pGPTV-Kan, containing the GUS gene downstream of the cloning site. The primers used for cloning are listed in S1 Table.

For SUS-RNAi lines, fragments (350–500 bp each) selected on the basis of lower sequence identity among the different genes were cloned from the 5' UTR of *SlSUS1* and from the coding sequences of *SlSUS3* and *SlSUS4*. The fragments (shown in S2 File) were then each subcloned in forward and reverse orientation into the pRNA69 RNAi cloning vector, which contains a plant-expression intron to create a stem-loop RNAi expression product [32]. The RNAi constructs were subcloned into the pGreen plant transformation vector [33] using the NotI restriction site. Primers for cloning are listed in S1 Table.

The constructs were transformed separately into electroporation-competent EHA105 *Agrobacterium tumefaciens* cells containing the pSoup T-DNA helper vector. The transformed *Agrobacterium* strains were used to generate transgenic tomato plant lines by cotyledon transformation [34]. R_0_ regenerants were cultivated from each transformation. Genomic DNA was assayed by PCR and transgenic plants were identified using the *nos* kanamycin-resistance gene. Confirmed transgenic lines were self-crossed to generate homozygous lines. The homozygosity of the F_2_ offspring was assessed using a TAQMAN real-time PCR assay [35].

### GUS staining

Histochemical localization of GUS activity was performed using 5-bromo-4-chloro-3-indolyl-b-D-glucuronide (X-gluc) as a substrate. Different tomato tissues were collected and placed in X-gluc buffer solution [0.75 mg/ml X-gluc, 50 mM NaPO_4_ (pH 7), 0.1 mM K_3_Fe(SCN)_6_, 0.1 mM K_4_Fe(SCN)_6_, 1 mM EDTA, 20% methanol] under vacuum at room temperature for 5 min and incubated overnight at 37°C. After incubation, the tissues were cleared with 70% ethanol and visualized under a binocular microscope. Freehand cross-sections were taken from stained stems and observed under transmitted white light. Digital images were taken using a CCD camera DC2000 (Leica, Germany).

### Carbohydrate-level assays

Samples (0.5 g each) of columella tissue from immature green tomato fruits collected from the first and second inflorescences (approximately 20 days post-anthesis, each fruit 2 to 3 cm diam.) or lateral leaflets of mature adult leaves (fifth to seventh from the apex) were collected and placed immediately in 80% ethanol. Soluble sugars were extracted in three consecutive soakings at 70°C. The ethanol from the three soakings was pooled for each sample and was then evaporated overnight at 55°C. Sugar residue was then dissolved in 1 ml H_2_O and filtered through a 0.25-μm micropore filter. Sucrose, glucose and fructose levels in the soluble sugar extracts were resolved by high-pressure liquid chromatography (HPLC).

The plant tissue remaining after this extraction was dried overnight at 60°C and then soaked in 6 ml H_2_O and heated to 121°C in a pressure autoclave for 1 h for starch extraction [36]. Extracted starch was broken down by overnight incubation with 10 mg/ml b-amyloglucosidase (Sigma) at 55°C. The levels of resulting glucose were measured using a Sumner assay.

### Fruit-set and seed weight measurements

For the fruit-set assay, five plants from each line were potted and grown in a greenhouse on the ARO grounds at ambient temperature. Side shoots on the plants were clipped, leaving a single growth axis. Fruit set was measured as the ratios of mature and developing fruits to the number of flowers counted on each plant.

For the average seed weight measurement, fifty seeds from four individual fruits were counted and weighed, and that weight was divided by the number of seeds.

### Gene-expression analysis

Shoot apexes, immature green fruits (about 2 cm diam.) and mature leaves from control and transgenic plants were flash-frozen and homogenized in liquid nitrogen. RNA was extracted using the EZ-RNA kit (Biological Industries; Bet Haemek, Israel) according to the manufacturer's instructions, with 100 mg homogenate used per sample. RNA pellets were dissolved in 24 μl DEPC-treated H_2_O with 3 μl DNAse buffer, 1 μl ribonuclease inhibitor and 1 μl RQ1 DNAse (Promega; Madison, WI, USA) and incubated for 1.5 h at 37°C. The reaction was halted by adding 3 μl 20 mM EDTA and then incubated 15 min at 65°C. The absence of DNA was confirmed by PCR with primers for actin, with samples as the template. Samples with no PCR product were used for the expression analysis.

To generate cDNA from the RNA samples, 1 μg of RNA was mixed with 0.2 μg of random hexamers (Sigma) and 0.5 μg oligo-dT, and the mixture was brought to a volume of 13 μl. The mixture was incubated for 5 min at 70°C and then for 5 min on ice. Five μl of 5x MMLV RT buffer, 1.25 μl dNTPs (10 mM), 0.5 μl RevertAid MMLV reverse transcriptase (Fermentas) and DEPC-treated water were added to bring the mixture to a final volume of 25 μl. The reaction mix was incubated for 50 min at 25°C and then for another 50 min at 50°C. The reaction was halted by incubation at 75°C for 15 min.

Real-time PCR reactions were carried out in a RotorGene 6000 cycler (Corbett Research; Mortlake, New South Wales, Australia) in a 10-μl reaction mix consisting of 4 μl cDNA, 1 μl of 10 pmol primers and 5 μl SYBR Premix Ex Taq II (Takara Bio Inc.). The reaction cycle began with 10 s at 95°C, followed by 40 cycles of 95°C/5 s– 60°C/25 s. The reaction ended with a gradual melt from 65° to 95°C. Results were interpreted using RotorGene software. The primers used are listed in S1 Table.

### Statistical analysis

All statistical analyses of expression data, fruit-set and seed weight measurements, and starch and sugar content were carried out using the JMP 5.0 software platform (SAS Institute; Cary, NC, USA).

### PIN1 reporter crosses

The SUS-RNAi transgenic line, S1R4, was crossed with a tomato line expressing the Arabidopsis PIN1 protein fused to the green fluorescent protein under the control of the native Arabidopsis PIN1 promoter (AtPIN1::AtPIN1:GFP) [37]. The F_1_ progeny were self-crossed and F_2_ segregants were selected for GFP expression and fluorescence, for SUS-RNAi expression and for S1R4 morphological phenotypes. F_2_ plants that retained GFP fluorescence, but did not express SUS-RNAi served as controls.

Shoot apices of S1R4/PIN1-GFP and control plants were collected and visualized under a fluorescence binocular microscope. Digital images were taken using a CCD camera DC2000 (Leica, Germany).

### RNA seq data analysis of SlSUS gene expression

RNA seq expression data were obtained for different organs of cultivar M82 [38], specifically meristems and primordia at different maturation stages [39], and viewed using the eFP browser (http://bar.utoronto.ca/efp_tomato/cgi-bin/efpWeb.cgi) and the Cold Spring Harbor Laboratory eFP browser (http://tomatolab.cshl.edu/efp/cgi-bin/efpWeb.cgi) [40]. Data were exported into an Excel spreadsheet and presented as expression in reads per Kb per million mapped reads (RPKM).

## Results

### Characterization of the tomato SUS gene family

Three SUS genes, *SlSUS1 SlSUS3* and *SlSUS4*, were identified before the publication of the tomato genome sequence and, following the release of the tomato genome sequence, we identified three additional SUS genes. The three new tomato SUS genes were also reported by Qin et al. [12] and named *SlSUS5*, *SlSUS6* and *SlSUS7*, bringing the total number of SUS genes in tomato to six, as in Arabidopsis. The SUS genes show similar characteristics in terms of genomic and cDNA length, as well as similar protein characteristics (as shown in Table 1).

**Table 1:** Characteristics of sucrose synthase genes in tomato.

**gene ID**	**name**	**reference**	**gDNA (bp)**	**cDNA (bp)**	**amino acids**	**MW (kDa)**	**PI**
Solyc12g009300	SUS1	Goren et al 2011	3945	2418	805	92.51	5.94
Solyc07g042550	SUS3	Goren et al 2011	5606	2822	805	92.59	5.96
Solyc09g098590	SUS4	Goren et al 2011	5365	2611	812	92.92	5.91
Solyc07g042520	SUS5	Qin et al 2016	3783	2847	803	91.63	5.97
Solyc03g098290	SUS6	Qin et al 2016	4210	2849	891	100.75	5.87
Solyc02g081300	SUS7	Qin et al 2016	4288	2955	884	100.69	8.42

To better characterize the different tomato SUS genes, we analyzed their genomic structures by aligning the genomic DNA sequences with the cDNA sequences using the BioEdit sequence alignment editor [41]. The six tomato SUS genes share a similar structure with nearly identical exon lengths (S1 Fig). While the translation of *SlSUS1*, *SlSUS3* and *SlSUS4* starts at the second exon, yielding large 5' UTR introns that are thought to be involved in gene regulation, *SlSUS5*, *SlSUS6* and *SlSUS7* do not contain any such introns (S1 Fig). The *SlSUS6* and *SlSUS7* genes are a bit longer than the others and have more exons at their ends, yielding higher molecular weight proteins of about 100 kDa as compared to the 92 kDa protein SUS1-5 (Table 1).

**Fig 1:**
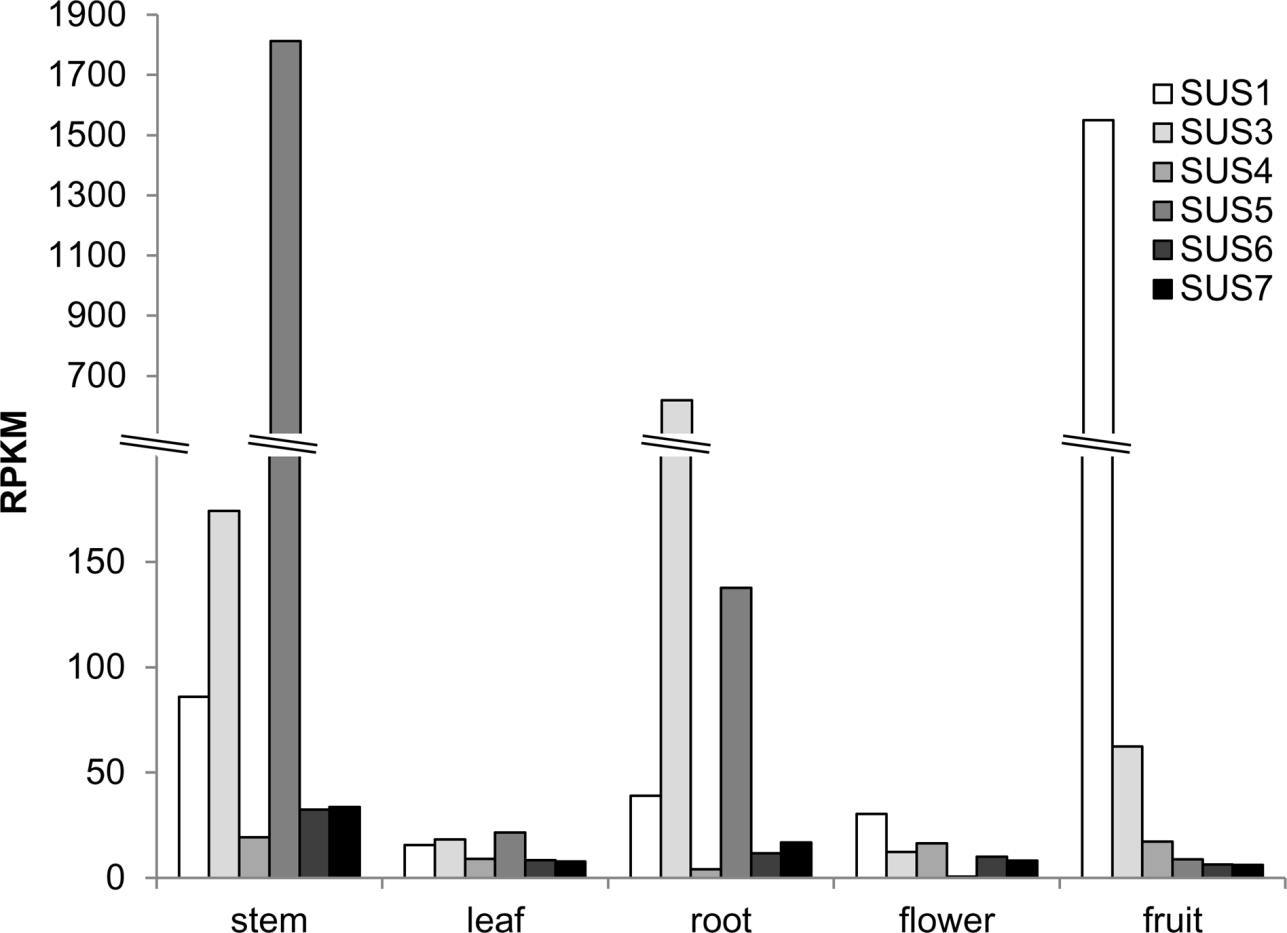
*SlSUS* genes relative expression in different organs. *SlSUS* gene family expression data obtained from RNA seq in different organs of the M82 cultivar. RPKM, reads per kilobase per millions mapped reads.

In terms of *SlSUS* gene expression, we looked at RNA seq data obtained in studies comparing gene expression in cultivated tomato with that of wild species [38]. The expression pattern of the SUS gene family in the M82 cultivar indicates that *SUS1* is the most abundant SUS expressed in fruit, *SUS3* is the most abundant SUS in roots and *SUS5* is the most abundant SUS in stems (Fig 1). In addition, there is also some expression of *SUS1* and *SUS3* in stems. In roots, there is some expression of *SUS1* and *SUS5* and, in fruits, there is some expression of *SUS3* (Fig 1). In leaves and flowers, the expression of genes from the SUS family is relatively low (Fig 1).

### The *SlSUS1*, *SlSUS3* and *SlSUS4* promoters drive GUS expression in differing patterns in the tomato plant

To further explore the expression patterns of the *SlSUS* genes and to resolve those patterns at the tissue level, we generated transgenic lines expressing the β-glucorinidase (GUS) reporter gene under the control of the promoters of *SUS1*, *SUS3* and *SUS4*, which were the only known SUS genes prior to the publication of the tomato genome. We cloned the three promoters from ~1000 bp upstream of the transcription start site (TSS) to the start codon of each gene (S1 File). The cloned regions included the large 5' UTR introns, which are likely to be involved in gene regulation [31, 42]. More than three independent lines were created for each proSlSUS::GUS construct. One line with relatively high GUS expression was chosen for further analysis using GUS staining of different tissue samples.

**Fig 2:**
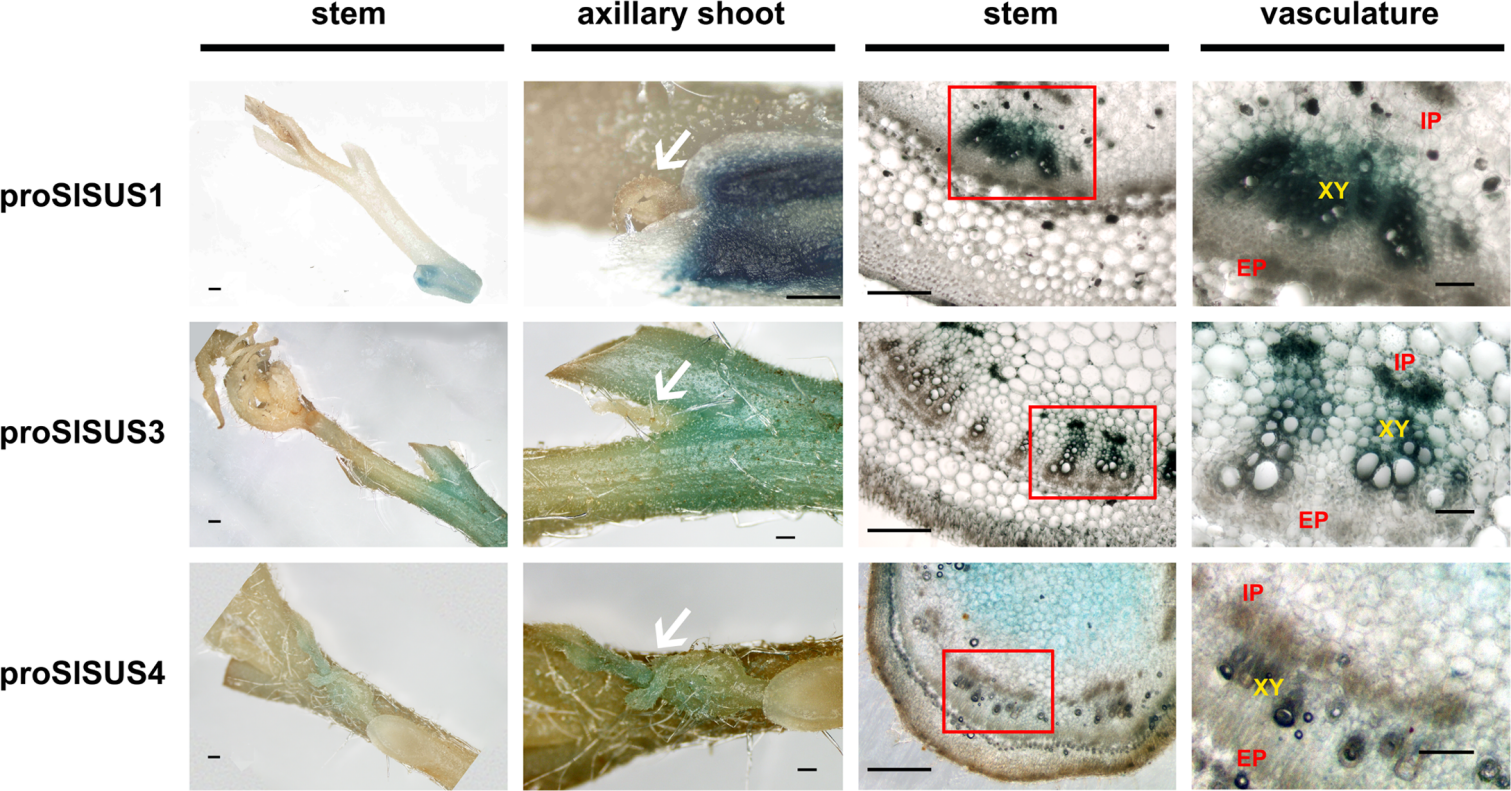
Expression patterns of the three *SlSUS* genes in young stems. Comparative GUS stains of stems from tomato lines expressing GUS under the control of each of the three *SlSUS* promoters (*proSlSUS*). Leftmost column: whole stem segment, bar– 1 mm; second column from left: magnifications of leftmost panels, showing axillary shoots (arrows), bar– 0.5 mm; third column from left: micrographs of stem cross-sections, bar– 0.5 mm; rightmost column: magnification of boxed areas from previous panels; EP–external phloem; IP–internal phloem; XY–xylem vessel members; bar– 0.1 mm.

The three promoters displayed differing patterns of expression that were easily discernible in the GUS stains (Fig 2). *proSlSUS1&3*::GUS stained prominently in the lower, more mature parts of the stem and that staining was restricted to the vascular tissue; whereas *proSlSUS4*::GUS stained the younger tissues, such as the axillary buds (Fig 2, white arrows), and that staining was located chiefly in the parenchyma of the stem. Interestingly, *proSlSUS1*::GUS staining was centered mainly in the xylem tissue; whereas *proSlSUS3*::GUS staining was concentrated specifically in the internal phloem tissue (Fig 2). These patterns were observed in the petioles as well (S2 Fig).

**Fig 3:**
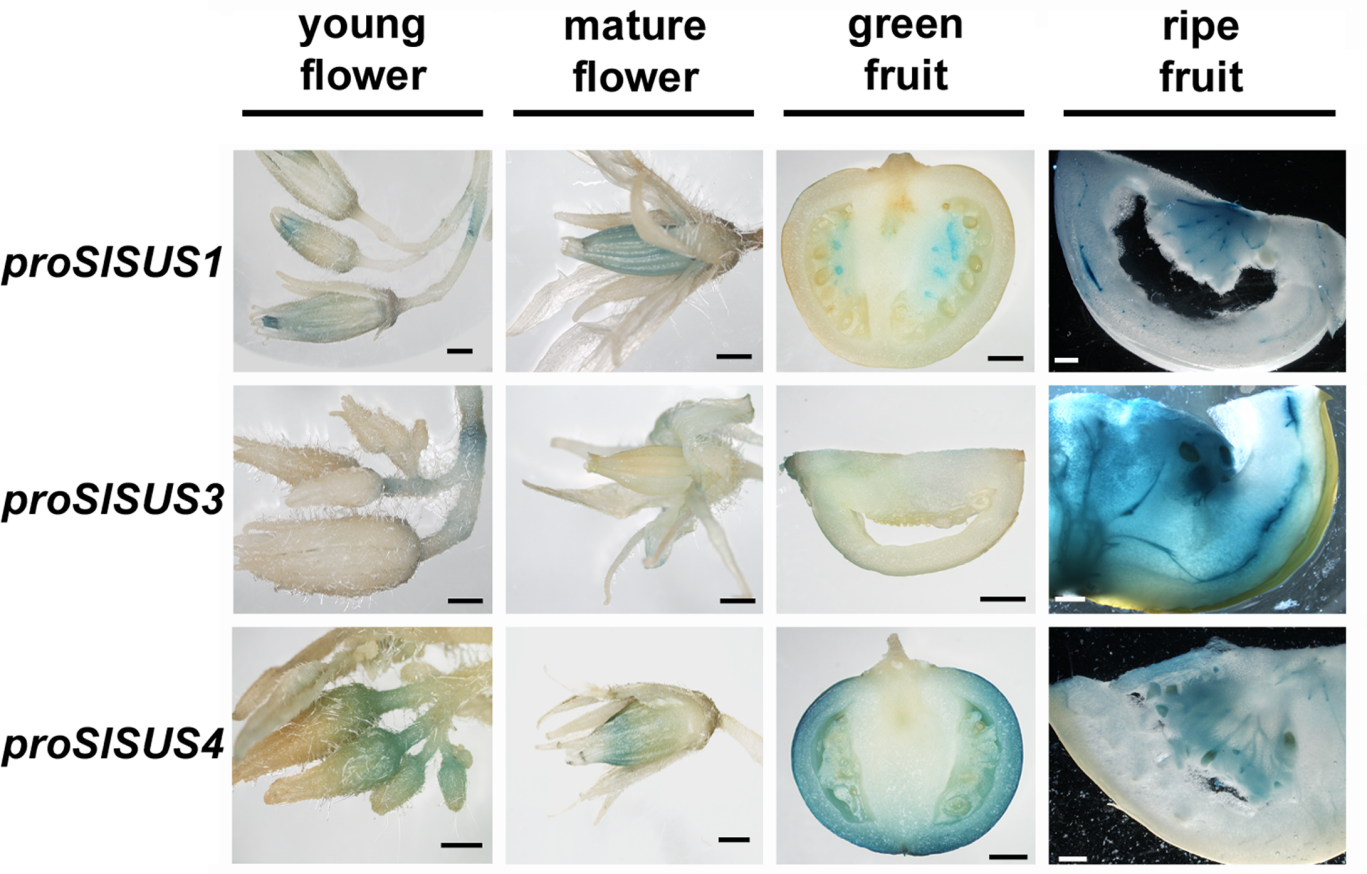
Expression patterns of the three *SlSUS* genes in reproductive organs. Comparative GUS stains of flowers and fruit from tomato lines expressing GUS under the control of each of the three *SlSUS* promoters (*proSlSUS*). Leftmost column: flowers before anthesis; second column from left: flowers at anthesis; third column from left: ~1 cm green fruit; rightmost column: ripe fruit; bar– 2 mm.

Differing expression patterns were also discernible in the flowers and fruits (Fig 3). *proSlSUS1*::GUS specifically stained young and mature anthers, as well as the abscission zones of each flower; whereas *proSlSUS3*::GUS stained the abscission zones exclusively. For both of these promoters, staining in the floral abscission zones was restricted to vascular tissues (S3 Fig). *proSlSUS4*::GUS displayed a changing pattern of expression over flower maturation, staining the base of immature flowers and the anthers and pistils of mature flowers (Fig 3).

We have previously shown that *SlSUS1* is expressed at its highest levels in young tomato fruit [10]. However, GUS staining revealed that *proSlSUS1* activity is limited to the columella tissue at the center of the fruit (Fig 3). In contrast, *proSlSUS4*::GUS, which stained the base of the flowers and its ovules, also strongly and specifically stained the pericarp of young fruit. *proSlSUS3*::GUS stained young fruit only weakly (Fig 3). All three *SlSUS* promoters demonstrated a shift in activity pattern in mature fruit, with both *proSlSUS1&3*::GUS staining concentrated in the vascular network throughout the fruit; whereas *proSlSUS4*::GUS stained the region surrounding the maturing seeds, as well as the seeds themselves (Fig 3).

**Fig 4:**
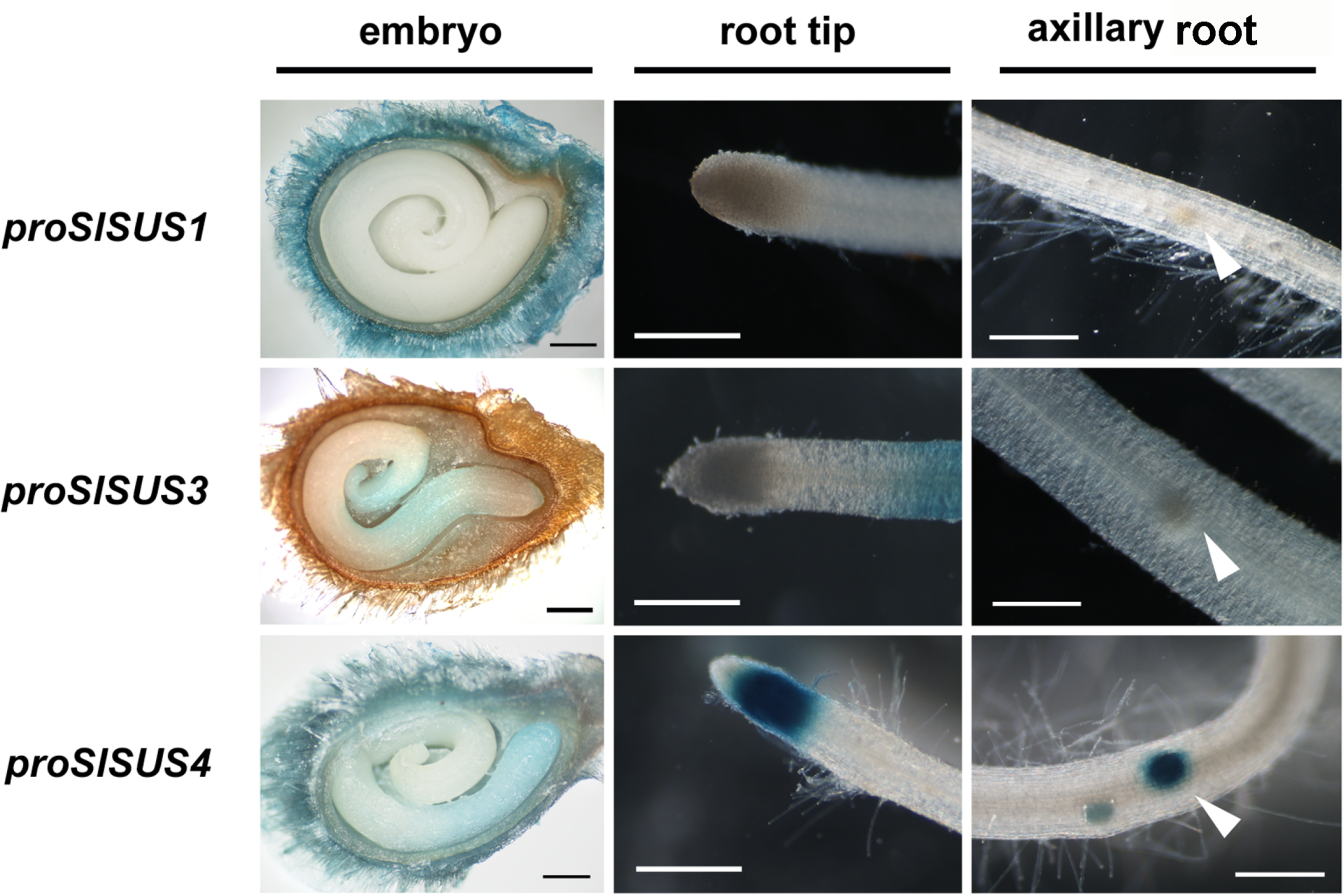
Expression patterns of the three *SlSUS* genes in embryos and seedling roots. Comparative GUS stains of germinating seeds and seedling roots from tomato lines expressing GUS under the control of each of the three *SlSUS* promoters (*proSlSUS*). Leftmost column: cross-sections of ungerminated seeds; center column: radicle tips of 3-day-old seedlings; rightmost column: seedling roots, showing the initiation of root branches (arrowheads); bar– 0.5 mm.

The specific activity of *proSlSUS4* in young, proliferating tissues was apparent in the seeds and seedling roots of the GUS reporter lines (Fig 4). *proSlSUS1*::GUS did not stain the embryo or seedling roots at all. *proSlSUS3*::GUS activity was detected in the region of the embryo corresponding to the center of the seed, comprising the elongation zones of both the root and the cotyledons, as well as in seedling roots well above the tip region (Fig 4). *proSlSUS4* activity was noticeable specifically in the root tip of the embryonic and seedling radicle, not including the root cap (Fig 4). In addition, GUS activity was observed in axillary root initiation points along the length of the seedling roots (Fig 4).

### Abnormal cotyledon morphology in *SlSUS*-RNAi lines with suppression of more than one SUS gene

**Fig 5:**
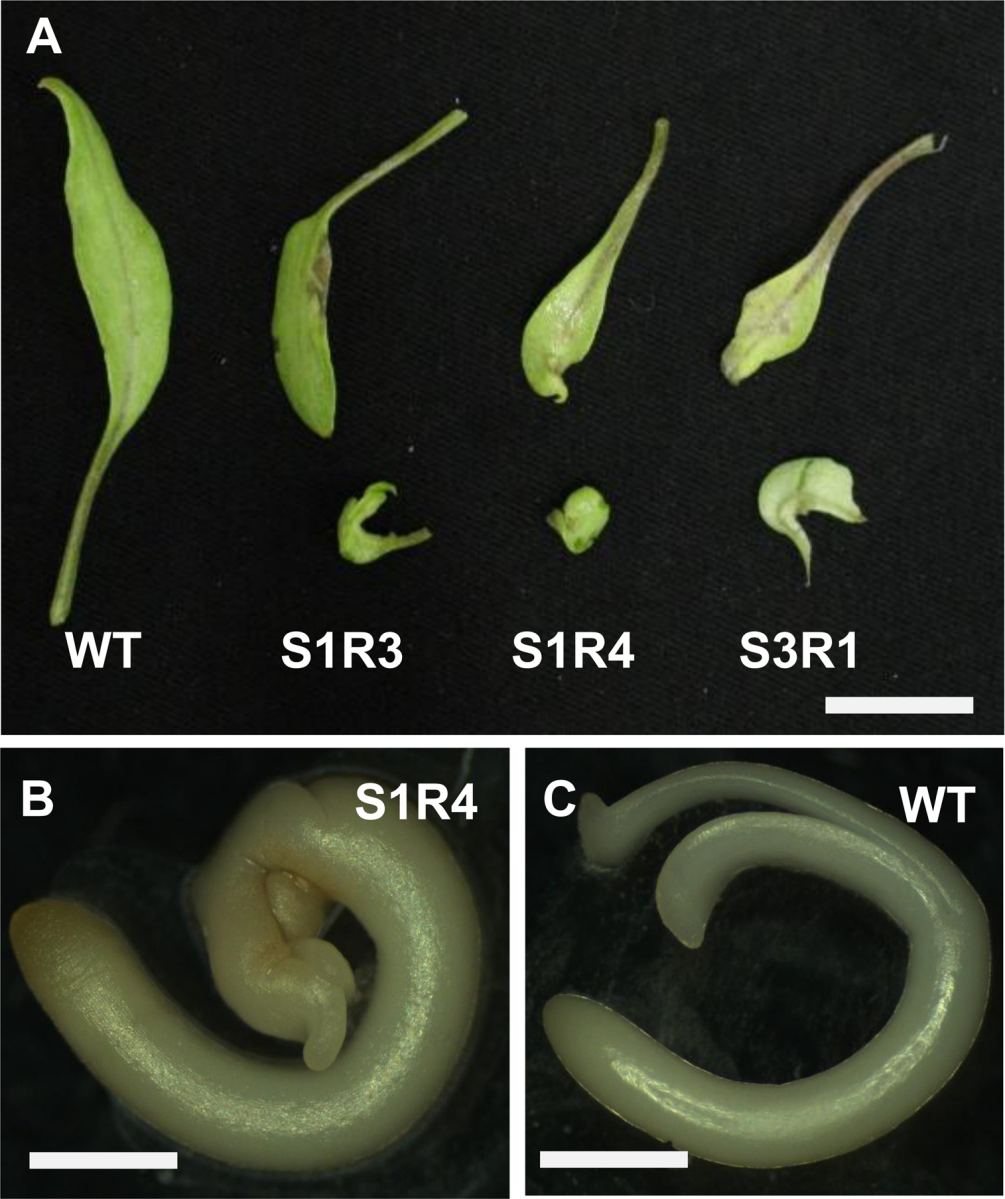
*SlSUS-RNAi* lines exhibit abnormal cotyledon morphology. **(A)** Cotyledons from wild-type (WT) seedlings and seedlings of three *SlSUS-RNAi* lines. Each pair of cotyledons was taken from a single seedling. Bar– 1 cm. **(B)** S1R4 embryo and **(C)** WT embryo extracted from seeds soaked for 24 h. The warped cotyledon of the S1R4 embryo is clearly visible; bar– 0.5 mm.

**Fig 6:**
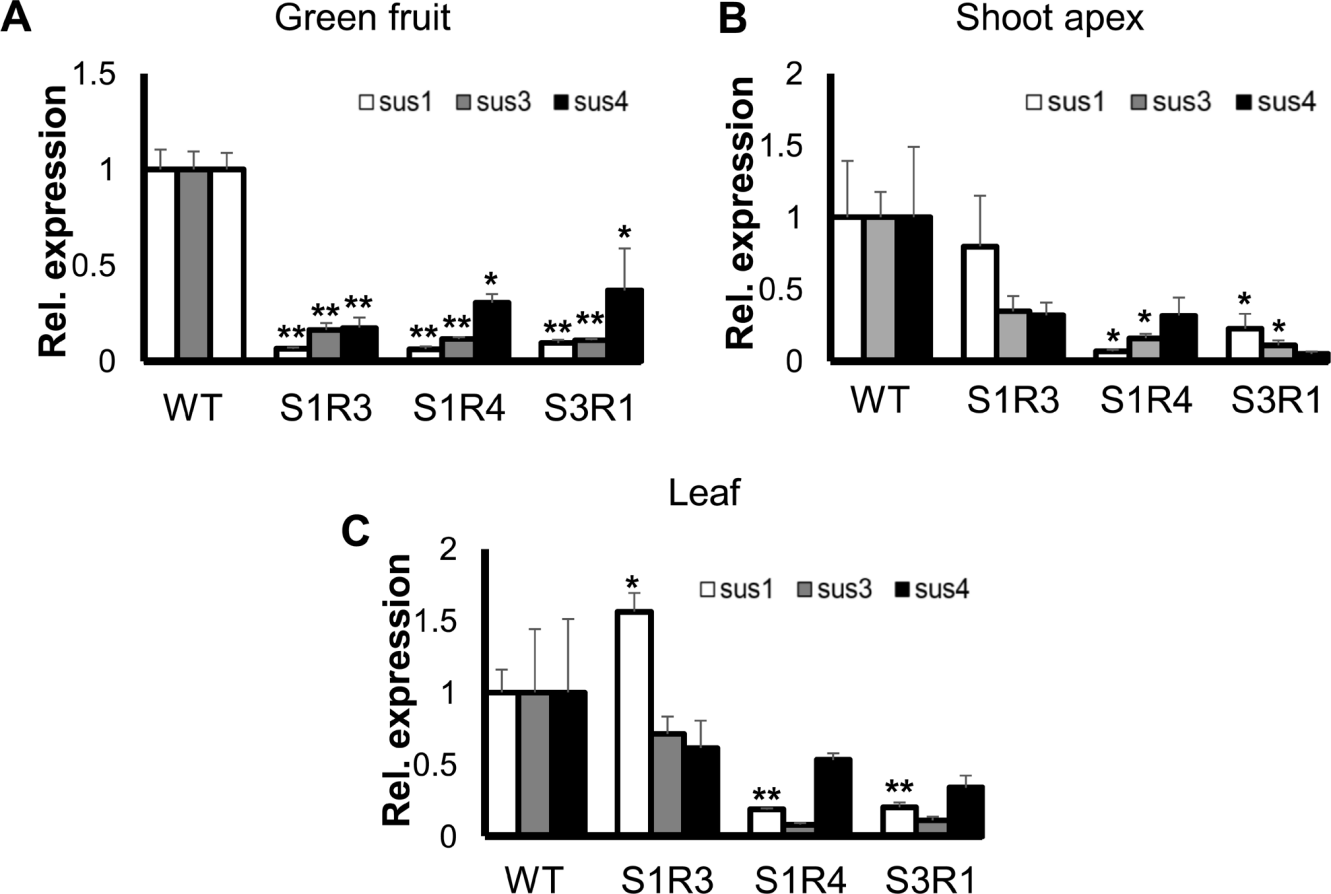
*SlSUS-RNAi* lines exhibit *SlSUS* co-suppression. **(A-C)** Reduced expression of *SlSUS* genes. RNA was extracted from green fruits, shoot apices, and mature leaves of *SlSUS-RNAi* and WT lines. cDNA was generated and subjected to real-time PCR analysis, using primers specific for *SlSUS1*, *3* and *4*. Cyclophilin was used as a reference gene. Error bars indicate the standard error (*n* ≥ 3). Asterisks indicate a statistically significant difference from the WT (* *P* < 0.05; ** *P* < 0.01).

To better elucidate the roles of *SlSUS* genes in development, we next set out to modulate expression of these genes by means of RNA interference (RNAi). We generated transgenic tomato plants containing RNAi constructs targeting *SlSUS1*,*3&4* genes (SUS1-RNAi, SUS3-RNAi & SUS4-RNAi). At least three independent, transgenic SUS-suppressed lines were identified for each construct. Among the transgenic lines generated, three in particular stood out due to abnormalities in the size and shape of one or both cotyledons of each seedling (S4 Fig). Two of these lines, S1R3 and S1R4, carried the construct targeting *SlSUS1*, and one, S3R1, expressed the construct targeting *SlSUS3*. The abnormal cotyledons appeared dwarfed and displayed a severe lateral curl, usually associated with the loss of bilateral symmetry (Fig 5A). Abnormality of the cotyledons could be observed in embryos prior to germination (Fig 5B and 5C). On external examination, the abnormalities appeared to stem from a lack of blade growth on one or both sides of the midrib. Due to the abnormalities in the shape and size of their cotyledons, Lines S1R3, S1R4 and S3R1 were selected for further analysis. The three lines, S1R3, S1R4 and S3R1, exhibited a more than 80% reduction in *SlSUS1&3* relative expression, as well as a significant reduction in *SlSUS4* expression in their green fruit (Fig 6A). Similar patterns of *SlSUS* co-suppression were also observed in the shoot apices and leaves of S1R4 and S3R1 transgenic lines (Fig 6B and 6C). Although the RNAi constructs were designed for specific gene suppression, some lines, including S1R3, S1R4 and S3R1, exhibited co-suppression of *SlSUS* genes. The most probable explanation for this is the high level of sequence identity among the conserved regions of the *SUS* genes. Most likely, the RNAi of one transcript caused the degradation of related gene transcripts and smaller nucleic-acid fragments with high identity to other SUS genes caused the degradation and suppression of other SUS genes.

### Transgenic lines with suppressed *SlSUS* show reduced fruit-setting and seed weight

**Fig 7:**
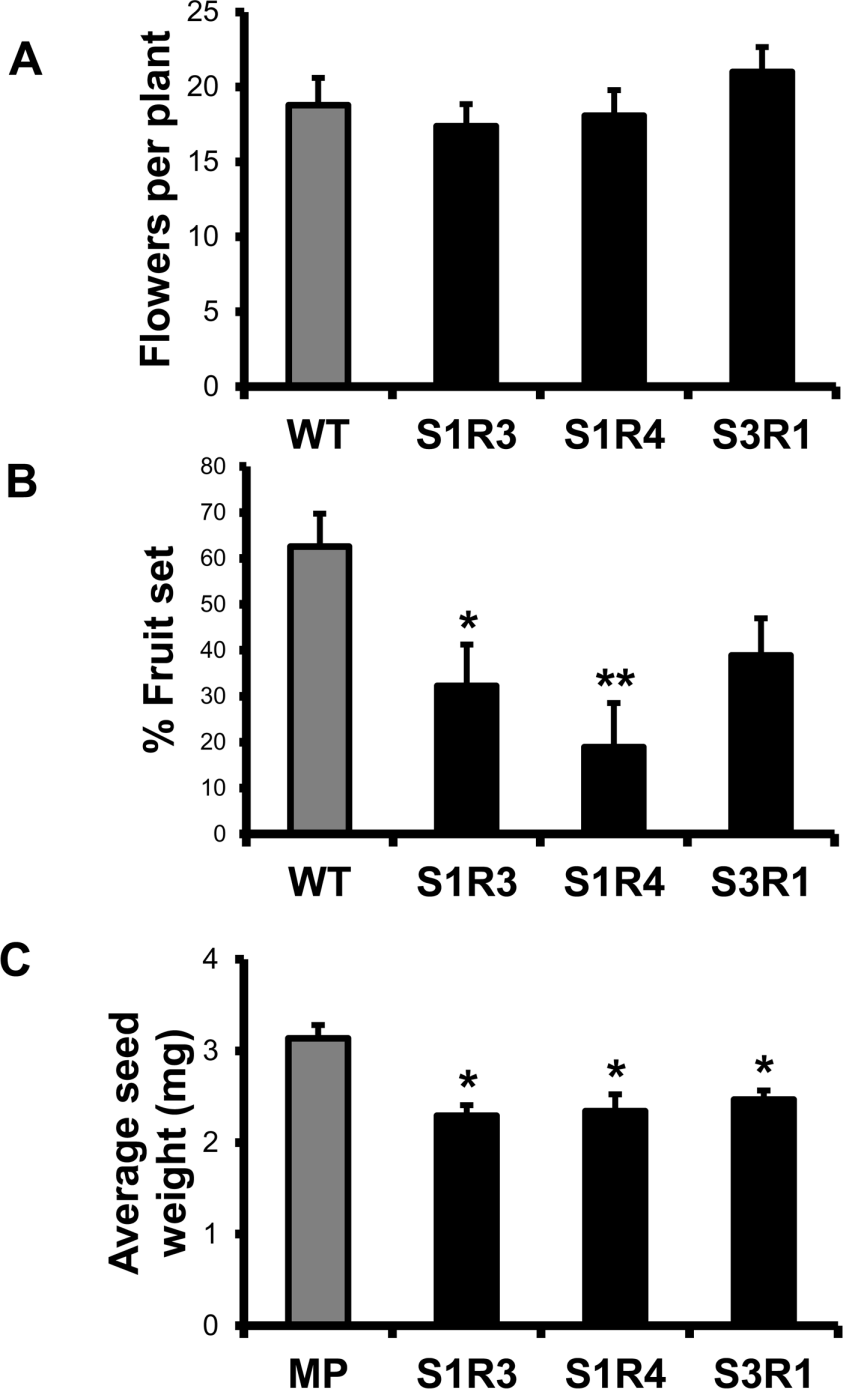
Effect of *SlSUS* suppression on tomato fertility and seed viability. **(A)** *SlSUS-RNAi* and WT plants were grown in a greenhouse to maturity. Flowers from the first five inflorescences from the ground of each plant (*n* = 9) were counted. **(B)** Fruit set was calculated as the number of fruit divided by the number of flowers from the first five inflorescences of each plant (*n* = 9). **(C)** To calculate average seed weights, 50 seeds per fruit (*n* = 4) were counted and weighed, and that weight was divided by the number of seeds. Error bars represent the standard error. Asterisks indicate a statistically significant difference relative to the WT (* *P* < 0.05; ** *P* < 0.01).

Despite the fact that there were no significant reductions in the number of flowers per plant (Fig 7A), the three transgenic lines (S1R3, S1R4 and S3R1) showed reduced fruit-setting (defined as percentage of flowers generating fruit; Fig 7B), a phenotype reported in a previous study of SUS-antisense tomato plants [29]. Similarly, seed weight was reduced in these lines relative to the control plants (Fig 7C). These additional phenotypes were not apparent in lines in which only one or two *SlSUS* genes were suppressed.

### Abnormal leaf morphology of the *SlSUS*-RNAi lines

**Fig 8:**
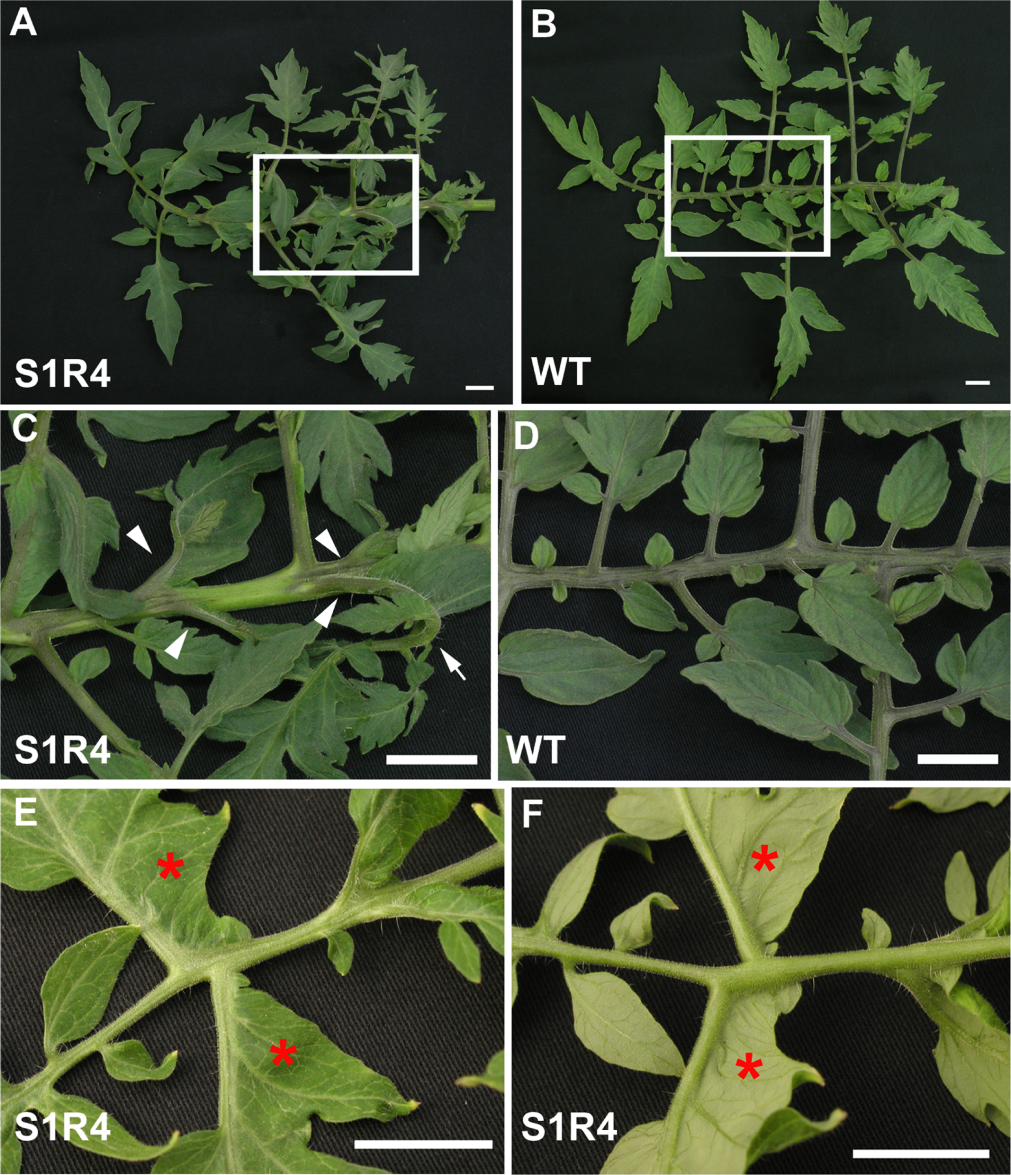
The S1R4 line exhibits abnormal leaf morphology. **(A)** Mature S1R4 leaf, **(B)** mature WT leaf, **(C)** magnification of the boxed areas in the S1R4 leaf, **(D)** magnification of the boxed areas in the WT leaf and **(E)** close-ups of the adaxial and **(F)** the abaxial side of an S1R4 leaf, showing ectopic blade formation of the leaflets (red area). Note the angle of the petiolules (arrowheads) and of the leaflet curling (arrow). Bar– 2 cm.

Line S1R4 also displayed abnormalities in the morphology of its leaves. These abnormalities were seen only in adult leaves (i.e., from about the 5^th^ or 6^th^ true leaf of the plants; Fig 8A and 8B). In the abnormal leaves, the rachis was curled abaxially and laterally. The leaflets and lobes were angled both adaxially and proximally (toward the petiole) and were often curled as well (Fig 8C), relative to the wild type (Fig 8D). The curling and angling were more pronounced toward the proximal part of the rachis. In addition, the leaves exhibited ectopic blade outgrowth, particularly in the region of the leaflets and lobes proximal to both the petiole and the rachis (Fig 8E and 8F). Similar, but less extreme abnormalities were observed for Line S1R3 (S3 Fig). Despite the obvious deformities in the leaf structure of the *SlSUS*-RNAi lines and the aforementioned localization of *SlSUS1&3* expression to the vascular tissue, there were no discernable differences in the structure of the vascular tissue in the petioles of those leaves (S6 Fig).

### *SlSUS*-RNAi lines show no changes in their starch and sugar levels

**Fig 9:**
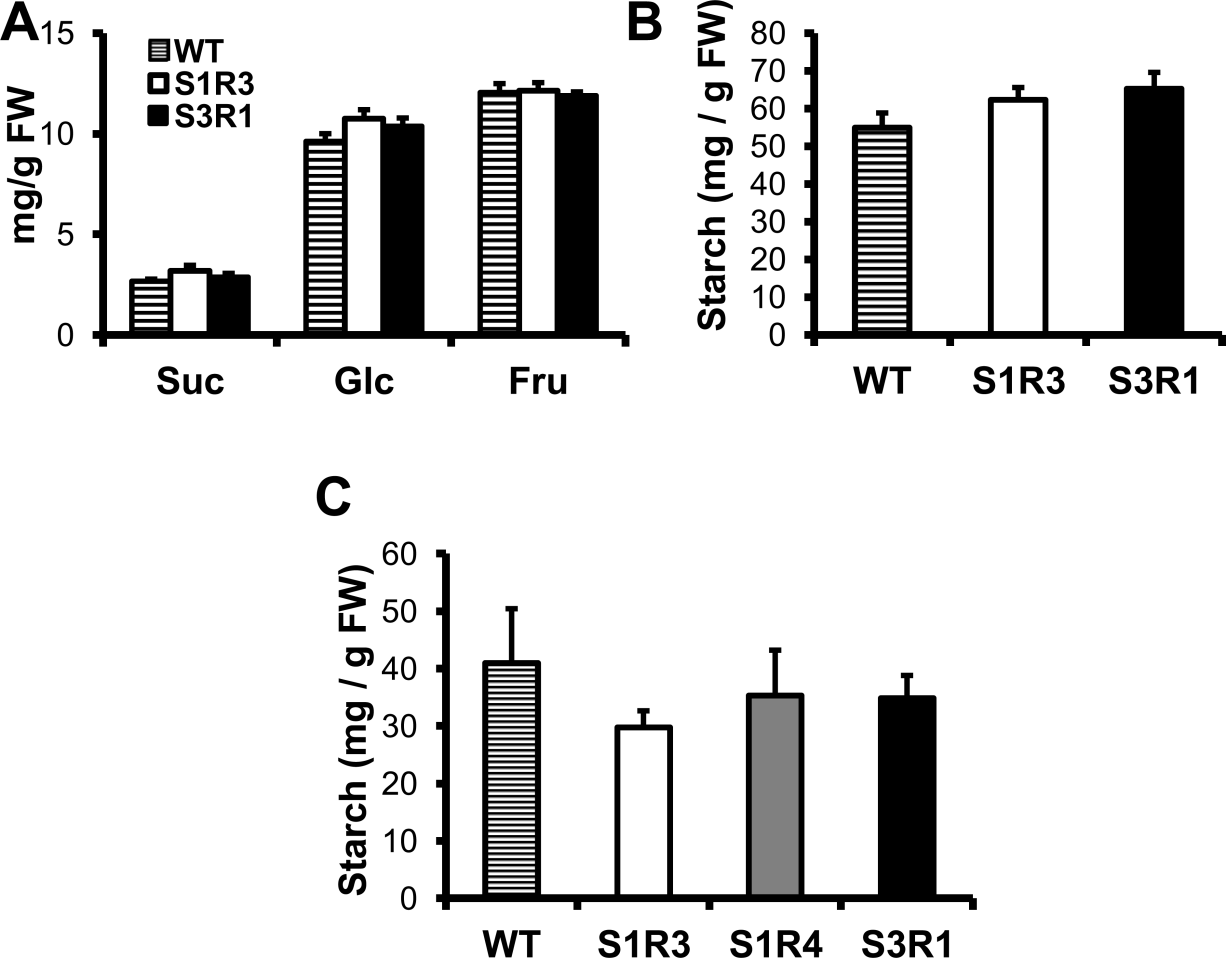
*SlSUS* suppression does not significantly affect soluble sugars or starch content. **(A)** Sucrose, glucose and fructose contents of young green fruit. **(B)** Starch content of young green fruit. **(C)** Starch content of mature leaves. Error bars indicate standard error (*n* = 5).

As SUS is considered to be primarily a metabolic enzyme involved in sucrose breakdown, starch synthesis and sugar-partitioning in plants, we next examined whether the *SlSUS*-RNAi plants showed any differences in their sugar levels, particularly in the abnormal leaves. Due to the aforementioned reduction in fruit-setting, there were insufficient fruits from Line S1R4 for this assay. Therefore, only leaf starch levels were measured for that line. Surprisingly, the sugar content of the transgenic fruits was not significantly different from that of the wild-type fruits (Fig 9A). Additionally, the starch contents of both green fruits and leaf tissue, including leaves with abnormal morphology, were not significantly altered in *SlSUS*-RNAi transgenic plants (Fig 9B and 9C).

### *SlSUS*-RNAi lines show altered expression of genes associated with leaf morphology and auxin levels

**Fig 10:**
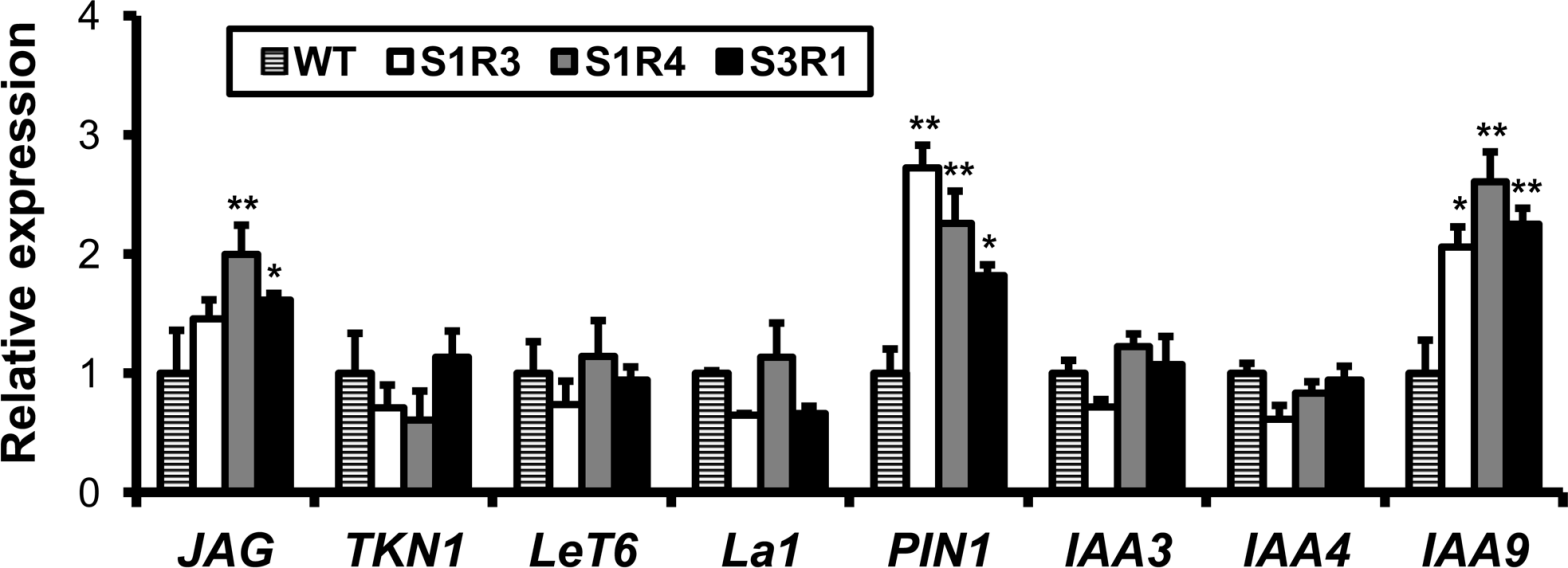
Suppression of SUS alters the expression patterns of genes involved in auxin signaling and leaf morphology. Relative expression of genes involved in the regulation of leaf morphology (JAG, TKN1, LeT6, La1) or auxin transport and signaling (PIN1, IAA3, IAA4, IAA9) as determined in the shoot apex. Cyclophilin was used as a reference gene. Error bars indicate the standard error (*n* = 3). Asterisks indicate a significant reduction relative to the WT (* *P* < 0.05; ** *P* < 0.01).

To further understand the altered leaf morphology, we measured the expression of genes associated with the abnormal leaf morphology in the *SlSUS*-RNAi transgenic shoot apices. Expression of the *JAG* gene, which affects the outgrowth of blade tissue in the tomato compound leaf [43], was significantly elevated in the transgenic lines (Fig 10). In contrast, expression of the transcription factors *TKN1* and *LeT6*, which affect leaf complexity, and of *lanceolate* (*La*), which affects leaf size, was not significantly altered (Fig 10). The *JAG* gene regulates blade outgrowth in leaf primordia via the auxin pathway, and auxin transport and accumulation are critical in the process of leaf patterning in tomato. We, therefore, measured the expression of auxin-related genes in the shoot apices as well. The transgenic lines had significantly elevated expression of the auxin transporter *PIN1*, which is involved in leaf patterning and blade outgrowth and which is also considered an indicator of auxin levels in plant tissue [37]. The auxin response factor *entire*/*IAA9*, which regulates blade outgrowth of leaflets, petioles and petiolules, was also significantly elevated in the *SlSUS*-RNAi lines (Fig 10). These findings indicate that altered *SlSUS* expression may affect auxin levels and signaling in transgenic plants.

**Fig 11:**
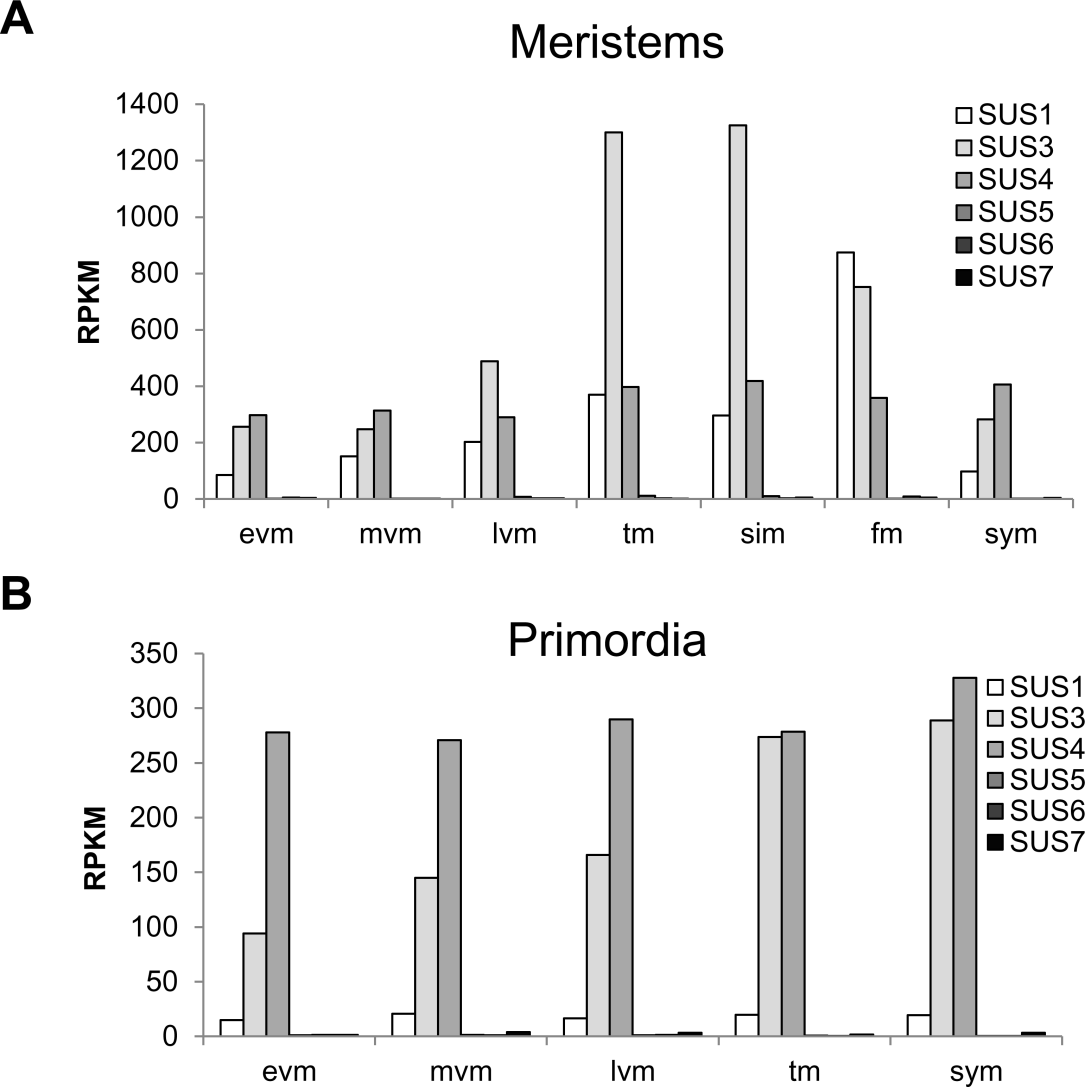
*SlSUS* gene family expression during meristem maturation. *SlSUS* gene family expression data from RNA sequencing performed for meristems **(A)** and primordia **(B)** of the cultivar M82 during meristem maturation (http://tomatolab.cshl.edu/efp/cgi-bin/efpWeb.cgi). Early vegetative meristem (evm), middle vegetative meristem (mvm), late vegetative meristem (lvm), transition meristem (tm), sympodial inflorescence meristem (sim), flower meristem (fm) and sympodial shoot meristem (sym) were analyzed.

To better understand which of the SUS genes might be involved in the altered leaf morphology phenotype we looked at the SUS gene family expression in RNA seq data obtained from tomato meristems and primordia during different meristem maturation stages (http://tomatolab.cshl.edu/efp/cgi-bin/efpWeb.cgi) [39]. Among the six SUS genes, *SlSUS1*,*3&4* are the main SUS genes expressed in the shoot apex and primordia in all stages, while the expression levels of *SlSUS5*,*6&7* are very low (Fig 11A and 11B). This expression pattern further support the importance of SUS1,3&4 in the determination of leaf morphology in the shoot apex.

### *SlSUS*-RNAi lines have altered distribution of the PIN1 auxin transporter in their developing leaves

To elucidate the effect of *SlSUS* suppression on PIN1 expression and auxin distribution in developing leaves, we crossed Line S1R4 with a transgenic tomato line expressing a fusion protein of the auxin transporter PIN1 and green fluorescent protein (GFP) under the control of the endogenic PIN1 promoter [37, 43]. Because S1R4 has the strongest leaf-morphology phenotype, it was the only line used for this cross, on the assumption that it would be the best system in which to detect any altered PIN1 distribution. However, the real-time PCR results (Fig 10) indicate increased expression of PIN1 (and other leaf-morphology genes) in the meristems of all three lines, suggesting similar effects. We then examined the shoot apices of the resulting S1R4/PIN1-GFP plants using confocal fluorescence microscopy and used a PIN1-GFP segregant that did not contain the RNAi construct as a control.

**Fig 12:**
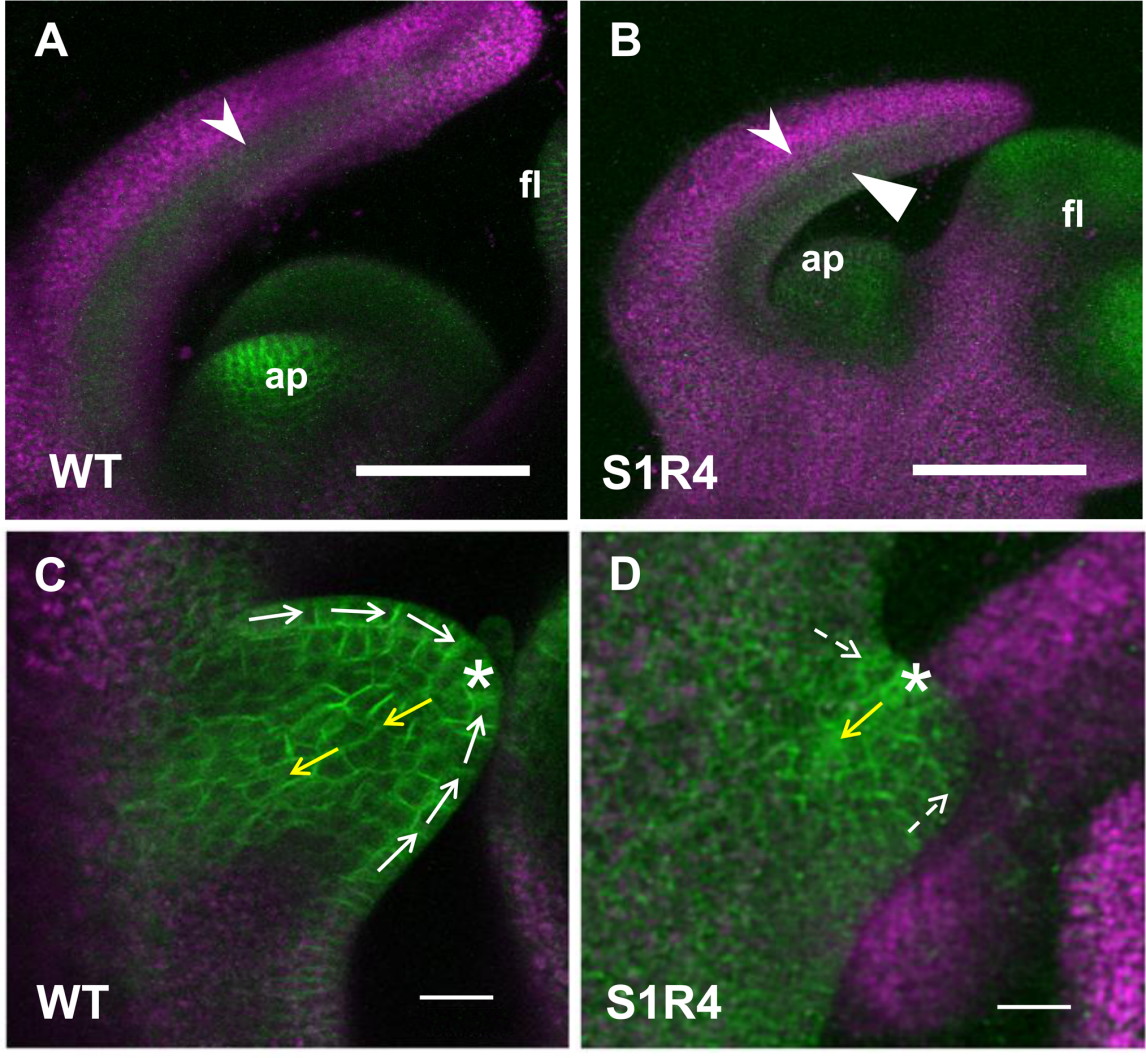
Auxin transport is altered in leaf primordia of SlSUS-suppressed plants. **(A, B)** Shoot apices and the youngest leaf primordia; ap–apex; fl–inflorescence primordium; indented arrowhead–normal PIN1 distribution along the center line of the primordium; flat arrowhead–ectopic distribution of PIN1 on the adaxial side of S1R4 primordium. **(C, D)** Leaflet primordia in older leaf primordia. Normal auxin transport proceeds along the periphery of the primordium (white arrows) with an auxin maximum at the apex (asterisk) and then flows through the center line (yellow arrows) along the path of the future vascular tissue. In the S1R4 primordium, transport is disorganized (dashed arrows with dashed lines) and the positions of the apex and centerline are altered. Bar– 100 μm.

In leaf primordia, young leaves and leaflets, the flow of auxin proceeds along the peripheral cells toward the future termini, then turns in toward the stem, traveling through the center of the primordia along the path of the future vascular bundle [44]. In the wild-type plants, the distribution of PIN1 in the leaf primordia fit this symmetrical pattern (Fig 12A, indented arrowhead). However, in the S1R4 PIN1-GFP hybrid, there was more fluorescence in the adaxial peripheral cells (Fig 12B, arrowhead). The S1R4 hybrid plants showed aberrant localization of PIN1-GFP in some of their leaflet primordia. The auxin maxima moved from the future leaf terminus to a more distal location (compare the asterisks in Fig 12C and 12D). The PIN1-GFP that turns toward the stem also moved from the center of the leaflet (Fig 12B, yellow arrow) to a more distal location (Fig 12D, yellow arrow). These observations further indicate that S1R4 plants have altered distribution of PIN1 in their developing leaves and possibly altered distribution of auxin as well, suggesting a mechanism for the altered leaf morphology of the SlSUS-RNAi lines.

## Discussion

As central as they are to plant life processes, sugars, in general, and sucrose, in particular, could be expected to be involved in regulatory mechanisms during growth and development. However, most research into sucrose-cleaving enzymes (sucrose synthases) in tomato and in other plants has focused on its metabolic roles, specifically cellulose synthesis [21, 27, 45] or starch synthesis [30, 46, 47]. In this study, we demonstrate for the first time a developmental role for SUS in tomato using SUS-RNAi lines in which multiple SUS genes were co-suppressed.

### *SlSUS* gene expression

We previously reported that the different isoforms of SUS in tomato are expressed in differential patterns, as assayed by real-time PCR, in which the differing patterns hint at coordination of the expression of the different isoforms, with a possible correlation to stem development [10]. Here, we investigated the expression patterns of *SlSUS1*,*3&4* by means of GUS reporter constructs, and report the expression pattern of the six SUS genes as extracted from RNA seq data [38, 39]. The RNA seq data suggest that SlSUS1 is the major SUS expressed in fruit, SUS3&5 are the major SUS expressed in roots, and SUS1,3&5 are the major SUS expressed in stems (Fig 1). In contrast, in leaves and flowers, there is relatively low expression of all SUS genes.

The GUS reporter lines provide a slightly better resolution and indicate that *SlSUS1&3* expression is focused in mature vascular tissues in the stem; whereas SlSUS4 was not detected in vascular tissues. The *proSlSUS4* GUS line mainly revealed expression in young proliferating tissues, such as the shoot apical meristem, young lateral shoots and main and lateral root meristems, as well as the radicle of germinating seeds and seedling roots (Figs 2 and 3). Our ability to observe expression in radicles and seedling roots was probably significantly enhanced by our use of GUS analysis, compared to RNA-seq data from an entire organ.

In green fruit, *proSlSUS4*::GUS expression was detected in the growing pericarp tissue; whereas in mature fruit, the seed envelope was stained. Expression restricted to these very small regions, easily masked in large tissue samples, could explain the drastic difference in the expression levels of *SlSUS4* relative to *SlSUS1&3* as measured by real-time PCR [10]. Although the promoter-driven GUS expression may help us to differentiate between gene-expression patterns within tissues, that assay can also be inaccurate. As seen in the RNA-seq data for the shoot apical meristems (Fig 11A), in tissues in which *SlSUS1*,*3&4* transcripts were present, only the promoter of *SlSUS4* showed GUS staining. A possible explanation for this is that not all of the regulatory elements promoting gene expression are located within the area that spans from a point located 1000 bp before the transcription start site to the translation site. A good example of that type of scenario is the tomato sucrose transporter 1 (*SUT1*), in which some of the regulatory sequences promoting expression in phloem companion cells, trichomes and guard cells are found in introns [48]

### *SlSUS* genes show redundancy

The different patterns and levels of expression of the *SlSUS* genes imply discrete roles for each gene. Nevertheless, all phenotypes and related changes in gene expression were observed only in transgenic lines in which there was significant down-regulation of *SlSUS1*,*3&4* together. These results could indicate that *SlSUS* genes nevertheless maintain a degree of redundancy.

Previous work with suppression of *SlSUS1* reported reduced fruit-setting when an antisense construct was expressed under a constitutive promoter [29], but not when it was expressed under a fruit-specific promoter [30]. The *SlSUS*-RNAi lines, which similarly used a constitutive promoter, also showed reduced fruit-setting, indicating that SUS activity contributes to the initiation of fruit in the flower, rather than in the developing fruit itself. Interestingly, as in those previous *SlSUS*-RNAi plants, the balance of sucrose, glucose and fructose, which could be expected to shift with a reduction in SUS activity, was unchanged in the transgenic fruit. This could mean that the low levels of *SlSUS* expression in the transgenic plants still provide sufficient SUS activity for metabolic purposes or that other metabolic enzymes are able to compensate for the loss of SUS activity. Alternatively, these results could imply that the effects of SUS reduction are more developmental than metabolic in nature. Arabidopsis plants with multiple *AtSUS* knockouts appear to develop normally and normal distributions of metabolites are seen in their mature seeds [15, 16]. There is still some debate as to whether this is a result of sufficient residual SUS activity in the knockout lines [46, 49] or whether SUS serves other roles in the plants.

### *SlSUS* suppression affects leaf morphology

*SlSUS*-RNAi lines demonstrated abnormal morphology of cotyledons and of mature leaves. The cotyledons appeared stunted and twisted, while mature leaves showed altered angling of the leaflets, and ectopic blade growth. Cotyledon morphology was already altered inside the seed, indicating that this effect represents impaired embryonic developmental effect.

The altered morphology of the mature leaves in the S1R4 lines and, to a lesser extent, the S1R3 lines, was manifested only in the adult leaves of the plants (i.e., from about the fourth or fifth leaf). A similar differential effect was reported in transgenic knockdown of the developmental gene *PHANTASTICA* in *Nicotiana sylvestris* [50]. In tomato, the juvenile leaves form on the shoot apex of the seedling very soon after germination and are probably subject to a different developmental program than the later-forming, adult leaves [51].

Leaf morphology and patterning are established in the leaf primordia and early stages of leaf development in the shoot apex. Analysis of the expression of the SUS gene family from RNA seq data from meristems and primordia during meristem maturation (http://tomatolab.cshl.edu/efp/cgi-bin/efpWeb.cgi) [39] indicated that among the six SUS genes, *SlSUS1*,*3&4* are the main SUS genes expressed in the shoot apex and primordia, while the expression levels of *SlSUS5*,*6&7* are very low (Fig 11A and 11B). These expression data support the roles suggested for SUS1,3&4 in the determination of leaf morphology in the shoot apex.

Suppression of another tomato metabolic enzyme, *SlFRK2*, which like *SlSUS1&3* is expressed in vascular tissue, leads to the wilting of leaves due to abnormal vascular development [52, 53]. However, no such abnormalities were observed in the vascular tissue of the *SlSUS*-RNAi lines, apparently ruling out vascular structure as the cause of the altered leaf morphology, in agreement with work showing that reduced SUS activity in stem vascular tissue does not affect plant growth or vascular tissue development in alfalfa (*Medicago sativa L*.) [54]. Instead, the altered morphology appears to stem from altered leaf patterning and blade outgrowth at the leaf primordia stage. The seemingly contradictory effects of insufficient blade growth in the cotyledons and ectopic blade outgrowth in mature leaves could further hint at the modulation of developmental signals regulating these processes.

### *SlSUS* suppression affects auxin signaling

A prominent signaling pathway involved in leaf patterning and blade outgrowth is that of the plant hormone auxin [37, 44]. Auxin is synthesized in apical meristems and leaf hydathodes and is transported symplastically toward the roots, forming a gradient that induces various signaling pathways in a concentration-dependent manner. Changes in auxin synthesis or transport can alter these signaling patterns. Auxin distribution patterns in the leaf primordia govern the structure of the compound tomato leaf and its pattern of blade growth [43]. The altered morphology of S1R4 leaves is somewhat reminiscent of the effects of the application of ectopic auxin to leaf primordia [44].

The changes in the expression of auxin-response genes in the shoot apices of our transgenic tomato lines, coupled with the altered fluorescence patterns of PIN1-GFP fusion proteins in leaf primordia of the S1R4/PIN1-GFP hybrid plants, indicate that auxin transport is indeed altered in these lines. The asymmetrical distribution of the auxin transporter PIN1 implies an asymmetrical distribution and flow of auxin in the primordia. As auxin in leaf primordia induces cell division [37, 44], this asymmetry could lead to asymmetrical growth of the leaf rachis, petiolules and blade tissue, as observed in S1R4 leaves. In addition, the higher expression levels of other auxin-associated genes along with that of PIN1, which is also considered to be an indicator of auxin levels [37], further suggest that total auxin levels may be ectopically high in the shoots of SlSUS-RNAi plants.

The gene *jagged* (*JAG*) regulates auxin synthesis during leaf morphogenesis [43]. This gene is significantly up-regulated in the *SlSUS* transgenic lines, implying altered auxin levels in these plants. Furthermore, the degree of *JAG* upregulation appears to be correlated to the severity of the morphological phenotype. The transgenic lines also showed increased expression of *IAA9*, an auxin-response gene involved in auxin signal suppression during leaf morphogenesis [55]. Knocking out *IAA9*, also known as *entire*, results in unrestrained blade growth, abolishing the compound structure of the tomato leaf [44]. While the ectopic blade outgrowth in S1R4 would seem more typical of *entire* down-regulation, the increased expression could represent an attempt at compensation for increased auxin levels resulting from altered distribution.

In addition to the effect of *SlSUS* suppression on auxin-related pathways and leaf morphology, it is highly possible that the altered cotyledon morphology is also auxin-related. Auxin has been shown to be one of the central players during embryo and cotyledon patterning [56–58] and several mutants that show altered polarity and distribution of auxin during cotyledon morphogenesis also show altered cotyledon numbers and the complete absence of cotyledons. For instance, the combination of pinoid with mutants of related kinases, auxin-synthesis genes, the NPH3-like gene ENHANCER OF PINOID (ENP) and PINFORMED1 (PIN1) itself results in cotyledon-less seedlings with variable penetrance [59–64]. Therefore, the phenotype observed in the cotyledons of *SlSUS*-suppression lines is likely to be auxin-related. In addition to the changes observed in leaves and cotyledons, some morphological changes were also observed in the roots of the S1R4 line, including a reduced number of root hairs and a thicker root at the early stages of germination. These effects, which are currently under investigation, might also be related to the auxin signaling pathway.

Further research is required to identify the link between sucrose synthase and auxin signaling. A possible connection could be an effect of cell wall cellulose content on partitioning and polarization of auxin transporters such as PIN1 [65]. As sucrose synthase is closely associated with cellulose synthesis and suppression of SUS has been reported to affect cellulose levels [21, 45], it is possible that suppression of *SlSUS* could affect auxin transport in the developing shoot apex and leaves by altering the structure and composition of the cell wall to which the auxin transporters are anchored [65]. However, if this is the case, it is less clear how this process might affect the expression of other auxin-related genes such as *JAG* and *IAA9*.

Another possible mechanism by which *SlSUS* suppression may affect auxin transport and signaling is by altering sugar signaling in the apical meristem, which, in turn, would alter auxin transport and signaling. There is evidence that sugar and auxin may co-regulate many genes. For example, in Arabidopsis roots, PIN1 is upregulated by auxin and by glucose and an additive effect is observed when roots are treated with both substances [66]. In a similar manner, transcriptome analysis of Arabidopsis roots identified 257 genes that are synergistically co-regulated by auxin and glucose. Those genes account for 68% of the genes in that transcriptome that were found to be regulated by either glucose or auxin [66]. Therefore, it is possible that *SlSUS* suppression in the shoot apical meristem leads to more sucrose degradation by invertase, yielding more free glucose, which may enhance the expression of auxin signaling and transport genes such as *PIN1*, *JAG* and *IAA9*.

### Concluding remarks

In recent years, evidence has accumulated to link sugar metabolism and developmental regulation. In pea plants, a model proposing coordination between sucrose accumulation and auxin signaling has been suggested in the context of apical dominance [67]. Sugars were reported to regulate vegetative phase change in Arabidopsis [68]. Trehalose metabolism, which is very similar to sucrose metabolism and is of regulatory significance in most plants, has also been implicated in developmental processes in the shoot apex, such as flowering regulation [69]. The effect of *SlSUS* suppression on leaf patterning could provide a logical link between the metabolic and hormonal pathways involved, though the exact mechanism remains unclear. Although sucrose synthase has long been considered an enzyme responsible for the synthesis and distribution of cellulose and starch, our results suggest that this enzyme also plays a role in plant development and morphology.

## Supporting information

S1 File Promoter sequences of the three SlSUS genes. The genomic sequence of the regions used for cloning the promoter sequences of the three SlSUS genes. Green boxed ATG–start codon; pink box–TATA box; blue text–exons; red text– 5’ UTR intron. Highlighted in yellow are the primers used for cloning the promoters.
(DOC)

S2 File Fragments used for creating the *SlSUS*-RNAi constructs. Highlighted in yellow are the fragments from each gene cDNA.
(DOCX)

S1 Table Primers used in this study. (XLSX)

S1 Fig Genomic structure of the *SlSUS* gene family. Comparative schematic presentation of genomic *SlSUS* sequences: *SlSUS5*, *SlSUS6* and *SlSUS7* genomic and cDNA sequences were obtained from the Sol Genomics Network (https://www.solgenomics.net/) and aligned with the *SlSUS1*,*3&4* gene structure described by Goren et al. [10]. Exons (*black*) have nearly the identical size in all isoforms, with the introns (*gray*) identically placed, but more variable in size. Numbers denote the size (bp) of exons (*horizontal*) and introns (*vertical*). ATG, start codon; TGA, TAA, TAG, stop codons.
(TIF)

S2 Fig SlSUS1,3&4 promoters expression in petioles. Free-hand cross-sections of GUS stained petioles observed under microscope (left column, Bars– 200 μm) or under dissecting microscope (right column, Bars– 500 μm); EP–external phloem; IP–internal phloem; XY–xylem vessel members.
(TIF)

S3 Fig SlSUS1&3 promoters are expressed in inflorescence abscission zones. **(A)** proSlSUS1 plants exhibit GUS staining in the inflorescence abscission zones. **(B)** proSlSUS3 plants exhibit GUS staining in the inflorescence abscission zones. **(C, D)** Longitudinal cross-sections of proSlSUS1 inflorescences show that GUS staining is primarily seen around the vascular tissues. **(A, B)** Bar– 1 mm; **(C, D)** bar– 100 μm.
(TIF)

S4 Fig Wild-type (WT) and *SlSUS-RNAi* cotyledons. Each pair of cotyledons was taken from a single seedling. Bar– 1 cm.
(TIF)

S5 Fig Abnormal leaf morphology of S1R3. Mature leaves of wild-type (WT) and S1R3 line.
(TIF)

S6 Fig Petioles of abnormally shaped leaves from S1R4 plants exhibit normal vascular structure. Light microscopy of free-hand cross-sections of S1R4 **(A)** and WT **(B)** petioles from mature leaves. Bar– 0.5 mm.
(TIF)
