# Supplementary material for: Suppression of sucrose synthase affects auxin signaling and leaf morphology in tomato
Source: PLoS One. 2017 Aug 7;12(8):e0182334. doi: 10.1371/journal.pone.0182334 (PMC5546705; doi:10.1371/journal.pone.0182334)
Supplement: S1 File — The genomic sequence of the regions used for cloning the promoter sequences of the three SlSUS genes. Green boxed ATG–start codon; pink box–TATA box; blue text–exons; red text– 5’ UTR intron. Highlighted in yellow are the primers used for cloning the promoters. (DOC) [file pone.0182334.s001.doc]

> SlSUS1 promoter and 5'UTR

ATATATAATGTTTGTTTGGTAAAAAATTGTCTTGTATTATAAGTGTATTAAAATGTGTGATAAATGTATTATCAATCATTAAAACTTGTATTATATTTGAATAATAAATTTATCTTTGTAATATATATTAAATTTGTATTATAAATGAATTAAAAATGGTCAAGTGAAAAAAAATGTTATTGCTATAAATGATAAATATTTCTTTATTATAGTATATTTATGTAAATTTTCCAAAAATAAAAGTCTCAATTTTGAGCAAGAAATACTAAATTTCTTTATTTTTATCCCTTTTTTTTATTATGATCATAATGTTCGGATTAATTTACATATTTCAATTAAAATAAATAATAATGAATAAACCTATACAATGGAGTATTTATAATTTATTGATCAAAATTTTAAAATTCACTTATTTTAAAAATGCTTTTTAGAAGTTTCTAAAAATTAGCGATTTGATGTTTGATTAATTTATCACTGACACAACAACTTAATCTTTAAATTATTTTTTAAAAATATATACTTATGACTTTAAAAAAGCTTGATTTAACCACCTAATTATATAATCAAAATTCTTACCATTGTATCAAATAATGTTTGTATAATTGAAAAAAAAATTGACCAGTCATTGACTGTTACACACTTTAACTTATCCAAATTAATAGTTAGATATTTCAATTTTGTAAAATACAAATATAAATATTTCAATTTGTTTTTACTATCTTTAAATAATTTGATTTATAAAAGAAAAAAATAATAAATATATTTCGAATTATCGTAAATGATTTGTAGATATATACTTTTGACAATGGATCACGTTGTCCAAAAATTAGAGTATATATATCGTTCACTCTTACTGAAGGATAAGTAGAAAAATCTTATTCGTTGATCCGATATTTAATAAATGTCGCCTAGGCGAATAAGACTATATCATGTGTTTATCTGTTAGTATATAGGTAGTTTTTGAACGGTAGAGACACCAATGTCCAAGAATGATAGGGGTATCTACATATCATTTATGATATTCAAAGATATATTATCCTTTTTTCTTAAAATAATAATTATTTAGACATTTTCAAAAATTTACGTGTAACAATAAAAATGATTTAATATGTTTACATATGTATTTTAAAAATTAACTTCGTTCAAATTGTCAACTGAGATCAAGTTAATAGGTTCAACTAGATATTGATTTTTTTTTCTTACTCACTAAACCAAAAGCCATCATTAGCATATAGTTTATGATTAATTAAGATCCTTCACCTAATTAATTAACACTTGCCACAATTTCCACTTTTTTTTGGCTATAAAAAGGTGTCCTCTAGCTTTGCTTTCTTCACCATTCACAAGCAAAACTCTTTCATTTGCTTCTTTCATTCATACACTCATTTCAACATTTTCTCCATTTTTTCTTTTTTCATTTCTTTCCTCTCAAAGgtaaagctacaattttttttaatttttttatatgctagttaatttccacatgcaaaaatagtcttttttttggtgtttggttcaaatttagttcaaagttttgatcttgggggaaaatgttgatcttatctttaaagggtgttaaagatcttgaatttttcttacagaaatttccattttaagttgagctcatttactctaaaacccagaaaaaagaagttattttttgatattttagctaaagattccaacttttttagtagtagaaagtgatttaagaacaaacaagaagtaatctttctggaaaaagttttgttcttgatcacccccaagaggtgtgaagtgttaaagatgacatttttgtggttttttttttttgaatttgtagaaagtgatcaagaacaaagtagaagtaatctttcttgaaaaagttttgttcttgaatcttgatcatgatcataatcaccccaagaggtgtaaagattacattttgaggtttgtttttgtgttcacatagtttttgtcacctttgtctcaaaactgtttttgttctgttctgttctgttctcatttgtttgtgggggtgggggtggggtggtcttgtttgttggtgtcaaaattcttagtttttaagtataccttcattttaagacaaatctatcttcatgacatagctgaagtagttcatgtttgctttagtcatcaattcttgttttgttttttcatagtacatttgctatttttctaatgaaaaacttacttggggttttcttaaagatcttgttttgttaagtttttaactaagatttgatgttttgattaaatcaagattgagaaatgtagtccattttgtaacagaaagtttactgtagattcttgttgtggggtcttcattgagttatttttttatatatatattttgtagtttagtctttgttgtagtcttggttgtaacgcataggcgttgtgcttttgccacaaacgtatttgagtaagaaatgtaactaatgtcatcctctttggttggttttggttttattagtactatagtattatactcagtagcggagccagaatttttagttaatgaattttgggtctagattccggatacattaaaaaaagaaaacaccctttagacgtattacagtagaagctttgtaggtagtagagtgttcagaatgctttgttatgtattttttcatggctcctttcactgtctttatttcttttttgaactgttttccttgagccgagggtctattggacgcaacttctctaccttcaaggtagaggtaaggtttgtggacattctaccctccccgacttcactttggtgagactacacggggtatgttgttggctccgcccttgactatattaggtgaaaggtatgaaaatttgaagtggtaacattgtggatgttgaaaatttaagtgattcagtgagttttttagtttgtcattttcaatttttcagtggttgtactaatgtaatgctatataaaattttgtttggtattcggataagctgaggtgggctcttctactaactgcaccagtgtatgttttggttgtttacagTTGAACTTTGTCTGAGGATTTCCCATCTGCTGAATCAACTATAATGGCTGAACGTGTTCTGACTCGTGTTCATAGACTTCGTGAACGTGTTGATGCAACTTTA

> LeSUS3 Promoter and 5'UTR

ATTGATTTACTATTTTATCCTTATATAAATATTAAATATTAATCTCTATATTTTATTCACTAGTGTTGTATCATTTAGCCTTCACTTCACATTGACTTTGAGTTTTGCAATTTCACATTATACTAAACTTCAAAACCTAAAGTAAGAAATATATTTCTCTTAATTTTTTCGTTGTGTTGCACTTTATTGTTTCATATTAGTTACAATTTTTTATATTAAATTAGTTACGTTATTATCTTGTCATATCAAATTATTGAAGATGTCATATATTTGTTTCATAAACATCTTCTTACTCATTAAATCTATAATTGAGCATTTAAATATCATATTTAATAAATCGATAAAATTATCTTATTGATTTATTGTATTTAAAAATTAAAAATCATAGCAACTGATATATAAAACAAAACAACCTATATATATCCCTATTTATCCTATATAAATCCTAATTAAACGAGCTTACAATATATACCAAAAGCTAGATAAATAAATAAAGTGATTAAGCACTATAGATATTATTTTCATGGTATTATTTTTCTGTCATGTCAAACCCAAGGTCTTTGAAAATGTGAACTTAGTTTTCTTTCTTTTCAAAAGAAGAAAATTGGACAAGAGGTAATTGGTTATTCAATGAACTCAAAGGCCAACAACTACCACAATTACTGTGAGATGAGATTTGTACCAAAAAAGTTAAATGGATAAATAATTTAAAGGTTTAAATAGTTATTAATTCTATATCATAAAGTTGAAGTGAATCTTAAAGTAAAAATAATTTTATCTTTACATAATTTATTAGTTATGAGTTGGAACTTAGAATAATAAGATAATATTTATCTAATAAGTGCAATCATTTCCTAAAATTTGCGTGAACAACATAAAGTACATCAAATTATCCTGTTTTGTAGGGTGTGTTTGGTAGTATGGAGGAAAGTTAACATTTTCTTATTTTCTTTTCCATGTTCAATTGATGAATCTTTTTGAAAAAAAATAAATTTTCTTAAAAAATAAAGATATATATCTTTTTCTTCCTGAATATAGAAAAAATAAATTTTATCAGTGACATTTTACATTGATCGTGATATTCTATCATTTTTTTTTATATCTTTAGCACACTTCATTTTTTATCCACATGCCTCAACTCTCATATTATTTATTTAAATACATAAAATATTTTAATATTATATTTTTTTCTTAGATATATCTCTTGTTTACTATTAAACATGATGAAATAAGAAATTTACTATTTTCGCATAAAAAAGTAATAAACATTTTTCTTGGATGGATACACCCATAATTATTAAATTATGTTTCTAAGAGAATTTTTTTTTTTTATCTTTTGTTATTATATTATAATTTAATTAATTTAAATTCGCGTTATATATATTAAGAATATTTTCATGTTTAATTTTAGAATCTAAAATCGATGAATAACTTTATTTATCACGTGAATCAGTGATTGTGATGGTGTTGCTTAAAAGAGCTTAGCAGGCAAACAAGAATTAATTAATATTCATAGCATTTTGTTTGTATAATTAAAATGTGATTCACTTGAGAAGACACGTACACATTTTGTATTTCTATAAAAAGGGACCTCTACCATTCCATTTTTTCTCATCACCATTCATAAGCAATACTCTTTCATTTCCATCTTTGTGTTAATTTCCTCCATCCATTACTTCCCTCTATTTATTATTATTCCTTTCAATATTCCTCTCTTTCCATTTTATTCATAAAAAATAAAAACTAAAAAAAGgtaaaacctgcgactctaatttgtttatgttatgtcggtgcgtaagaattttgttgtttcatccctatctcattcgtacatataaaaaaaaaggaataattttcagtagcatttgcttgcactactactcttgttcatgtacctttcgttttcagagtagttctttcttttttttttaacaaaaaaaaacattttaatattcatttttgttgactgataaaaagaagagattgtgagattttttttttgttatactctgcaaattctaactttactttcttttttttttcatttttttgacaatattgacagaaaaatctatttataaccgtcacggatagaaaacaatgtattatacatttgacgagaagaaacaaaaatcctcacatctcttttcatctttttctcctgttagtattgctctgtttttctttcctctgtttttttatttttttcaattctagctatttagatgttgctttgtgtggggttgaatgggttaaaatcattttatttttacttgatttagtttgacgtgagcttcagatatcgaatgattaaaaaaaatagtttccaatcaagacatcttttatcttcataatgttttagtatttgattttgtcacaatttctttaatttcttcccagttttttgttttcgagtgaaacgttgaggaaaacggggaaattttgttgatatttactaaattctagatggcatattctttcactttatttcttttgaaatataatatttgaagggtacctattgatcatatagtatatagggagtatgtcataggtgttttttatttatttattggttcaattattggtgtggtttaactaaaacagtgaaatacgactgctaacacctattttttaataaatatataataggaagatcagtgcaagttctctctgttgtgttattattgggccacatttcttttggtcgtttacgtatcctattctgcatttatttgataactctcatgacattataatattgggtaggtctgctagtttttattattccgtccgtgttagtttgatcggacgagttgatgaattaaaataaaaaatttatgattttaaattatcagttagatatttgaattgaaaaaacttataaaatataaaaaatactttttttttttggaaaatatagtagagctaggtggggttgaaaatttgtgctagtatattctagataattggaaaattaggtgattaattaggtgacacgatctcatctctttgaatagcagtaatgtgctaaaatatttttatgatttttgagttaacagtattttgtttgtttatttttacagTTGAAAGTCATCTGAGGATTTGCAGGTGCAATGGCTCAACGTGTTCTAACTCGTGTTC

> LeSUS4 promoter and 5'UTR

TTCGAGGATTGACATGATCCTGGGTGTTTTGGGATGGTCCACGGGCTGCAGTTGGTCAGAGAACAAACAGTTTCTAGAATGCTAATTTAGTGGCCTGGTGAAGAGTAGTTGCCCAGCTGTTGAATGCTTGGGGAAGAAAGTTTTGTTCAGTGAAACCTGAGTTCTGTTTGACAAGTATAGAAGCAAGCAATATAGGTTATATGGTGTAGGGCGACTGTATATAAAGGAAGAGTAGCAACCAGTTATACTTGTAAAGGAATTTTCAACTATACGAACTCCAACAAAAGTATGTTACAGTTATACAAAATAAATATATTAAATTATTTGTCACATCTATTTCTAATGTGGTATGGGTTTGACACAACGTCACCGTGCTCCTTTGCCTTCTGCGTCCCACCACCACCGCAAATTCCCAACAGAAAATGGTAAGAGTGCAGACATTGCAGTTGGCTATATATGCCAAGTTTGCATCAGTAGTTACAACGATACTGTTAGTACAGTATACAAATCGGATCTATTTTTGCCTTTATTTAACAACTGATACACTTCTCTAACAAAATCAGTTACGTTAAGCATGCGTTTAAAGCATAAGAGCAAGTCAAAGAAAATGATATAAGCAGATGAGTCTGTTGTATCAGACTTCCTATGGCTTGAATCTTGCATAGTCTAGCTCTATGCTGATCTGCTTGGACAAATCTTGCGCTCCACGCATGTTGGTTCGAAAAGGCAAATGTGGATTTCTTCAAATTCAAACGATGCCCAAAATGAGAAGTAGAGATTTTAAATCAAAAAGCTATCGAACAATCATATCACACAACTTTTTCAGTTCAAATAAGGCTCCGAAACAAAAAAAATAGGAATTTTCCAAAGGGGCAAATGTAGGCCCACGCCACATAGCTTGAAACTCATCCCACCCATTGTCATCCCCATGCATTAGTGTCACTAAATAAAAACGCATACTTAATTTTTCGAGTCTCAGCTCTGAAAATTTTAAATTGTAGTTTACTGATTCGATTTTGACTCTAAATTTTGTGAGTCTACTTGTGATAACCTAGTGAATGATATTTATTACTTCTAGAAGACCAACATCACTTTTTTATAGTTTCATGTCATAAAAATAAATTCAAACTTATCTCAAATTTTAGTGTTTCCATTTTTTTAACCCATCGTGCCAAGTGATGCACGCGTTGGAGGAACTACACCTGTGTTTGGTCTCTACATCGACGCTACAAGGATTGAGGGTACGGTGACAAATGGTTGGATTGATTCAGATTTTGATGGATTGAATATGAGTTGACCAAAAATGGTTTGGATTACAATCCACACAAATAAAATATGACAAATATGGATTTGATCAAATATGATTTGGATAAAATAGTTTCAACTTACCCCTTCCCCTCGGGCACCCCCTCTCCCCAGCCCTTCTCCCGACCTTCCACCAACCGACCCCTAAAAAACTTACCCACTCCTCGGGCACCCCTCCCGGCCTCCCCCAATCTTCCACCAACCTACCCCTAATTTTTTTTGTTTTCTAAAAAAAATAAAAAGAATCTGTTTTTCAAAAAGAGAAAAAGATTTAGCCCCTTCCTCCGTGCACCCTCAACCCCCAATCTTCCACCAACCTATCCCAAAATATTTTTGTTTTTTAAAAACAAAAAAACTCTATTTCATTTCACTTATACGAGCTGAATATAGATTCTCATGTTAACCCATTTTGTCCATATTTATATGAATTTAAAGAAGTTGAAAACCATATTAACTCTTTTATTTTTTTTAAAAAAGAAATTCATTTAATATGAGAAAACTTATTTCATTTGAGCTTGAATTGACATCCTCCAAGGATGAAATAAAGAAATTTTTTTTATTACCCGAAATCGACCGTTTTGGAATCCGTTGAATGgtaattactatatattgttcagcatgttattattattttcttttttagaacttttagcggacaaacacaaatctaatatagttacttacagctgcctaaagttatactgtgatttaggctactggtgttttccctccccaataaattgtcatccaagaatagtgaactataatatatgataagtacatattatgtggtatatagtaagattattttatttttaattagattttaaatttaaataatgaatataaaaaaaatgttattagaagcgtttgatcgaaagaaattttctcatgaatgaccaaaacattagattgacatgggatgaaataattctacagtccatttctggagggagataagaagtaggtttattttattcatttaataaataagttgatcgaaaatggcacgtgtagaaaaaaataatggacatttggcagagcttaaacaatcacattattataattgttattatattataggccgaaagtttggaatggacagatgtcaggtttaacaacatttgagctgcgaccacgacagcacaatgaatttattcaaaccacgatgatataacagaccagaagggaagaaaatatataaaataattagagataataatttctacacaattttagttctataaaaaaaaacttattataaataataataattagacatattcatgggtcactttctatattatattatattaaaaagaatttattaagaaggtggtgtattcagTATTCTATCTATTTTGTTGTTGTAATTGGAAAATGTCGAATCCAAAGTTGTCAAGAATACCTAGTATGAGAGAGAGAG

ATG – start codon TATA box EXON intron Promoter cloning primers
