# Supplementary material for: Suppression of sucrose synthase affects auxin signaling and leaf morphology in tomato
Source: PLoS One. 2017 Aug 7;12(8):e0182334. doi: 10.1371/journal.pone.0182334 (PMC5546705; doi:10.1371/journal.pone.0182334)
Supplement: S2 File — Highlighted in yellow are the fragments from each gene cDNA. (DOCX) [file pone.0182334.s002.docx]

SlSUS1 cDNA

AGAGGTGTAAAGATTACATTTTGAGTTGAACTTTGTCTGAGGATTTCCCATCTGCTGAATCAACTATAAT

GGCTGAACGTGTTCTGACTCGTGTTCATAGACTTCGTGAACGTGTTGATGCAACTTTAGCTGCTCACCGC

AATGAGATACTGCTGTTTCTTTCAAGGATTGAAAGCCACGGAAAAGGGATCTTGAAACCTCACGAGCTTT

TGGCTGAGTTCGATGCAATTCGCCAAGATGACAAAGACAAACTGAATGAACATGCGTTCGAAGAACTCCT

GAAATCCACTCAGGAAGCGATTGTTCTGCCCCCTTGGGTTGCACTTGCTATTCGTTTGAGGCCTGGTGTC

TGGGAATACGTCCGTGTGAACGTCAATGCACTAGTTGTTGAGGAGCTGTCCGTCCCTGAGTATTTGCAAT

TCAAGGAAGAACTTGTCGACGGAGCCTCAAATGGAAATTTCGTTCTCGAGTTGGATTTCGAGCCTTTCAC

TGCATCCTTTCCTAAACCAACCCTCACCAAATCTATTGGAAATGGAGTTGAATTCCTCAATAGGCACCTC

TCTGCCAAAATGTTCCATGACAAGGAAAGCATGGCCCCGCTTCTCGAATTTCTCCGCGCTCACCATTATA

AGGGCAAGACAATGATGCTGAATGATAGGATACATAATTCGAATACTCTTCAAAATGTCCTAAGGAAGGC

AGAGGAATACCTCATTATGCTTCCCCCGGAAACTCCATTTTTTGAATTCGAACACAAGTTCCAAGAAATC

GGATTGGAGAAGGGATGGGGGGACACGGCGGAGCGTGTGCTAGAGATGGTGTGCATGCTTCTTGATCTCC

TTGAGGCACCTGACTCATGTACTCTTGAGAAGTTCTTGGGGAGAATTCCGATGGTCTTCAATGTGGTTAT

CCTTTCCCCTCATGGATATTTTGCCCAAGAAAATGTCTTGGGTTATCCCGACACCGGTGGCCAGGTTGTC

TACATATTAGATCAAGTTCCCGCCTTGGAGCGTGAAATGCTTAAGCGCATAAAGGAGCAAGGACTTGATA

TCATCCCCCGTATTCTTATTGTTACTCGGCTGCTGCCCGATGCAGTTGGAACCACTTGCGGTCAGAGGCT

TGAGAAGGTGTATGGAACAGAACACTCACATATTCTTAGAGTCCCCTTTAGGACTGAGAAGGGTATCGTT

CGCAAATGGATCTCTCGCTTTGAAGTGTGGCCATATATGGAGACTTTCATTGAGGATGTTGCAAAAGAAA

TATCTGCAGAATTGCAGGCCAAGCCAGATTTGATAATCGGAAACTACAGTGAGGGCAATCTTGCTGCTTC

TTTGTTAGCTCACAAGTTAGGCGTAACGCAGTGCACCATCGCCCATGCCTTGGAGAAAACGAAATATCCT

GATTCCGACATTTACTGGAAAAAGTTTGATGAAAAATACCATTTCTCGTCCCAGTTTACAGCTGATCTCA

TTGCAATGAATCACACTGATTTCATCATCACCAGCACATTCCAGGAGATAGCAGGAAGCAAGGACACTGT

AGGACAATATGAGAGCCATATGGCATTTACAATGCCTGGATTGTACAGAGTTGTTCATGGCATTAATGTG

TTCGACCCCAAGTTCAACATCGTCTCACCTGGAGCTGATATTAACCTCTACTTCCCGTACTCCGAATCGG

AAAAGAGACTTACAGCATTTCACCCTGAAATTGATGAGCTGCTGTACAGTGACGTTGAGAATGACGAACA

TCTGTGTGTGCTCAAGGACAGGACTAAGCCAATTTTATTCACAATGGCAAGGTTGGATCGTGTGAAGAAT

TTAACCGGACTTGTTGAGTGGTACGCCAAGAATCCACGACTAAGGGGATTGGTTAACCTGGTCGTAGTTG

GCGGAGATCGAAGGAAGGAATCCAAAGATTTGGAAGAGCAGGCAGAGATGAAGAAGATGTATGAGCTAAT

AGAGACTCATAACTTGAATGGCCAATTCAGATGGATTTCTTCCCAGATGAACCGAGTGAGGAATGGTGAG

CTCTACCGATACATTGCTGACACTAAGGGAGCTTTTGTGCAGCCTGCATTCTACGAGGCTTTTGGTCTGA

CTGTTGTCGAAGCAATGACTTGTGGTCTGCCTACATTTGCAACTAATCACGGTGGTCCAGCTGAAATCAT

CGTTCATGGAAAATCCGGTTTCCATATTGATCCATATCACGGTGAGCAAGCTGCTGATCTGCTAGCTGAT

TTCTTTGAGAAATGCAAGAAAGAGCCTTCACATTGGGAAACCATTTCGACGGGTGGTCTGAAGCGCATCC

AAGAGAAGTACACTTGGCAAATCTACTCCGAAAGACTACTGACGCTGGCTGCTGTTTATGGGTTCTGGAA

ACATGTTTCTAAGCTTGATCGTCTAGAAATCCGTCGCTACCTTGAAATGTTTTACGCTCTCAAGTACCGT

AAGATGGCTGAAGCTGTTCCATTGGCTGCTGAGTGAATGAAGGTCTTCCTGTTTTTTCTTTGAATAAAAA

TGAAGTCTTTGACAAGTAGAGGCTTCTGTTTAGTGTTTCATTTCCATTTAGTATCCCCCCCCCCACCCCA

CCCCACCCCACCCCCTTTGCTTTATGTTGTATTTTTCATTTGGTCATGTCAATGTAGGTTGGAGAATTTG

AGCTGTTAGTAGTATGAATAAGAGATCAAAATTTCAATCTATAAGTTATCAAGAAACATTGTTTTGGTTT

GCCTGTTGTCA

Highlighted in yellow is the sequence used for creating the SUS1-RNAi construct

SlSUS3 cDNA

GGGTAGGTCTGCTAGTTTTTATTATTCCGTCCGTGTTAGTTTGATCGGACGAGTTGATGAATTAAAATAA

AAAATTTATGATTTTAAATTATCAGTTAGATATTTGAATTGAAAAAACTTATAAAATATAAAAAATACTT

TTTTTTTTTGGAAAATATAGTAGAGCTAGGTGGGGTTGAAAATTTGTGCTAGTATATTCTAGATAATTGG

AAAATTAGTTGAAAGTCATCTGAGGATTTGCAGGTGCAATGGCTCAACGTGTTCTAACTCGTGTTCACAG

TCTTCGTGAACGTCTTGATGCTACTTTGGATGCTCATCGCAATGAAATTTTGCTCTTTCTTTCAAGGATC

GAAAGCCACGGGAAAGGGATCTTGAAACCTCACCAGCTACTGGCTGAGTTTGAATCAATTCAGAAAGAAG

ACAAAGACAAACTGAATGATCATGCCTTTGAAGAAGTCCTGAAATCCACTCAGGAAGCAATTGTTTTGCC

CCCATGGGTTGCACTTGCTATTCGTTTGAGGCCCGGTGTGTGGGAATATGTCCGTGTGAATGTTAATGCT

CTTAGTGTTGAGGAGCTGACTGTGCCTGAGTTTTTGCAATTCAAGGAAGAACTTGTTAACGGAACTTCCA

GTGATAACTTTGTTCTTGAATTGGATTTTGAGCCCTTCACTGCATCATTTCCAAAACCAACCCTCACGAA

ATCAATTGGAAATGGAGTTGAATTCCTCAACAGGCACCTCTCTGCTAAAATGTTCCATGACAAGGAAAGC

ATGACCCCTCTTCTCGAGTTTCTTCGAGTTCACCACTACAATGGAAAGTCAATGATGCTGAATGATAGAA

TTCAGAATTTGTATACTCTCCAAAAAGTCCTGAGGAAGGCCGAGGAATACCTCACCACCCTTTCGCCAGA

AACTTCATACTCCTCATTTGAGCACAAGTTCCAAGAAATTGGCTTGGAGAGAGGTTGGGGTGACACCGCA

GAGCGTGTTCTAGAGATGATCTGCATGCTCCTGGATCTCCTTGAGGCTCCTGACTCATGTACTCTTGAGA

AGTTCCTTAGTAGAATTCCTATGGTTTTCAATGTAGTTATACTTTCACCTCATGGATATTTCGCCCAGGA

AAATGTCTTGGGTTACCCCGACACTGGTGGTCAGGTTGTCTATATTTTGGATCAAGTTCCTGCCTTGGAG

CGTGAGATGCTCAAGCGCATAAAGGAGCAAGGACTTGATATCAAACCGCGTATTCTTATTGTTACTCGGC

TTCTCCCTGATGCAGTTGGTACCACTTGTGGTCAGCGACTCGAGAAGGTATTTGGAACTGAGCATTCACA

TATTCTTAGGGTCCCCTTTAGGACTGAAAAGGGCATTGTTCGCAAATGGATCTCTCGTTTTGAAGTCTGG

CCATACATGGAGACTTTCATTGAGGATGTGGGGAAAGAAATAACCGCAGAACTGCAAGCTAAGCCAGATC

TTATTATTGGAAACTATAGTGAGGGAAACCTTGCAGCCTCCTTGTTGGCTCACAAGTTAGGTGTAACACA

GTGCACCATTGCTCATGCATTGGAGAAAACCAAATATCCTGATTCTGACATTTACTTGAACAAATTTGAC

GAGAAATACCACTTCTCAGCTCAGTTCACAGCTGATCTTATAGCAATGAATCATACTGATTTCATTATCA

CCAGCACCTTCCAGGAGATAGCAGGAAGCAAGGACACTGTTGGACAGTATGAGAGCCACATGGCCTTCAC

AATGCCTGGATTGTATAGAGTTGTTCATGGCATTGATGTGTTCGACCCCAAATTCAACATTGTGTCACCA

GGAGCTGATGTGAATCTCTATTTCCCATACTCCGAAAAGGAAAAGAGATTGACAACTTTTCACCCTGAAA

TTGAAGACTTGCTGTTTAGCGATGTTGAGAACGAAGAACACCTGTGTGTGTTGAAGGACAGGAATAAGCC

CATCATATTCACCATGGCAAGATTGGACCGAGTGAAGAACTTAACTGGACTTGTCGAGTGGTATGCTAAG

AATCCACGACTAAGGGAGTTGGTTAACCTTGTAGTGGTTGGTGGAGACCGAAGAAAGGAATCCAAAGACT

TGGAAGAGCAGGCAGAGATGAAGAAGATGTATGAACTTATAAAGACTCACAATTTGAATGGCCAGTTCCG

ATGGATTTCTTCCCAGATGAACCGCGTGAGGAATGGGGAACTCTACAGGTACATTGCTGACACAAGGGGA

GCTTTCGTGCAGCCTGCATTCTACGAGGCTTTCGGTCTGACTGTTGTTGAGGCCATGAGCTGCGGTTTGC

CTACATTTGCAACTAATCAAGGTGGTCCAGCTGAGATCATCGTTCATGGAAAGTCTGGTTTCCAAATTGA

TCCATACCATGGCGAGCAGGCTGCTGATCTCCTCGCTGAGTTCTTCGAGAAATGTAAGGTAGACCCTTCA

CATTGGGAAGCCATTTCCAAGGGTGGCCTTAAGCGTATACAGGAGAAGTACACATGGCAAATCTACTCCG

ACCGGCTGTTGACACTAGCTGCTGTTTACGGGTTCTGGAAGCACGTTTCCAAGCTTGATCGTCTTGAAAT

TCGTCGTTATCTTGAGATGTTTTACGCTCTCAAATTCCGCAAGCTGGCTGAACTTGTCCCATTGGCTGTT

GAGTAAATTGACAAAGAAGAGAAGGTTTCTGTCTGATTGTTATCCACATTGTCCTTTAGAAATTGTTTGC

CCCACATTTGTATCTGTTTGAGAACTTCATTGTCTTTTTCATTTGCCATTTTTCCCTTCTGTAGTCATGA

AGAGGATTGCAAATTTGACATTATGTAGTGTTAGTGTGAATAAAATATCAAATTTCAATCTACTCCATCC

ATCCTCATATTAGAATTGAATACCGTCTCTACCTTCACAAGTTAATGACAGAAATCTATGTACGTATCAA

CTTCTCTCGATTTTATTTATGA

Highlighted in yellow is the sequence used for creating the SUS3-RNAi construct

SlSUS4 cDNA

ATTAAGAAGGTGGTGTATTCAGTATTCTATCTATTTTGTTGTTGTAATTGGAAAATGTCGAATCCAAAGT

TGTCAAGAATACCTAGTATGAGAGAGAGAGTTGAGGATACTCTCTCTGCTCACCGTAATCAGCTAGTGGC

TCTCCTATCCAGATATGTGGCGCAGGGGAAGGGGATATTGCAGCCTCATCATTTAATTGATGAGCTGAAT

AATGCTGTATGTGATGACACTGCTTGTGAAAAGCTGAAAGAAGGCCCCTTTTGTGAAATCTTGAAATCTA

CTCAGGAAGCCATTGTGCTCCCACCATTTGTTGCTATAGCAGTTCGTCCAAGGCCAGGTGTTTGGGAATA

TGTTCGTGTGAACGTATATGATCTGAGCGTTGAACAATTGACGGTTCCTGAATATCTCCGTTTCAAAGAA

GAACTTGTCGATGGAGAGGATCATAATCATCTTTTTGTGCTTGAGCTGGATTTTGAGCCATTTAATGCAT

CAGTTCCTCGTCCCTCACGCTCTTCATCCATTGGAAATGGAGTACAATTCCTCAATCGTCATTTGTCCTC

AAATATGTTTCGCAGCAACGAATCCCTCGACCCATTACTTGATTTCCTTAGAGGACACAATCATAAAGGG

AATGTCTTGATGTTGAATGAACGTATACAGCGAATCTCCAGGCTGGAGTCGTCTCTTAATAAGGCAGATG

ATTATCTCTCAAAGCTACCACCAGATACACCCTATACTGACTTTGAATATGCATTGCAGGAAATGGGCTT

TGAGAAAGGTTGGGGTGATACTGCAAATCGTGTTTTGGAGACTATGCATCTGCTTTCTGACATTCTTCAG

GCTCCTGATCCCTCAACCTTAGAGACTTTTCTTGGTAGATTACCAATGGTGTTTAATGTTGTCATATTAT

CTCCTCATGGATACTTTGGTCAAGCAAATGTCTTGGGTTTGCCGGACACTGGTGGACAGGTTGTCTATAT

ACTGGATCAAGTGCGTGCTTTGGAGGCCGAAATGCTTCTTAGAATAAAGCAACAAGGACTCAACTTCAAG

CCTAGAATCCTTGTTGTCACCCGACTGATACCTGATGCTAAAGGAACCACGTGCAACCAGCGGTTGGAGA

GAATTAGTGGAACTGAATACTCGCATATTTTACGTGTCCCTTTTAGGACAGAAAATGGAATCCTTCATAA

ATGGATATCTAGGTTTGATGTATGGCCTTACCTGGAGAAGTTTACTGAGGACGTGGCAGGTGAAATGTCT

GCTGAGCTCCAGGGAGTGCCTGATCTGATTATTGGCAACTACAGTGATGGAAATTTAGTTGCCTCCCTGT

TAGCATATAAAATGGGCATCACACAGTGTACCATTGCTCATGCTTTGGAGAAAACAAAGTATCCTGATTC

TGACATCTATTGGAAAAAGTTTGAGGAGAAATATCATTTTTCATGTCAGTTTACTGCTGACCTACTGTCA

ATGAATCATTCAGATTTCATAATCACTAGTACCTATCAAGAGATTGCAGGAACGAAGAATACTGTTGGTC

AGTACGAGAGTCATACTGCTTTCACTCTCCCAGGCCTATATCGTGTTGTCCATGGCATTGATGTCTTCGA

TCCCAAATTCAATATAGTGTCTCCTGGAGCTGACATGACAATTTACTTCCCATATTTTGACAAGGAAAAG

AGACTGACATCTTTGCATCCCTCGATTGAGAAGTTGTTATTTGATCCTGAGCAGAATGAAGTGCATATAG

GCAGCCTGAATGATCAATCAAAACCGATAATTTTTTCAATGGCAAGGCTTGACCGGGTAAAGAACATTAC

CGGATTAGTTGAGTGCTATGCTAAAAATGCCACACTCAGGGAACTGGCTAACCTTGTTGTAGTAGCTGGA

TACAACGATGTAAAGAAATCCAATGATAGAGAAGAAATAGCAGAAATTGAGAAGATGCATGCTCTTATGA

AGGAACATAACTTGGATGGTCAATTCAGATGGATATCAGCCCAAATGAACCGGGCACGTAATGGTGAGCT

CTATCGCTATATAGCTGACAAGAGAGGTATATTTGTTCAGCCTGCGTATTATGAAGCGTTTGGACTGACG

GTGGTTGAAGCTATGACTTGTGGTCTTCCAACATTTGCAACTTGTCATGGTGGACCTATGGAGATCATTC

AGGACGGTGTATCCGGGTACCATATAGATCCTTATCATCCCAATAAAGCTGCTGAGCTCATGGTAGAATT

CTTCCAACGATGCGAACAAAATCCTACTCACTGGGAAAATATATCTGCATCTGGCCTACAAAGGATTCTC

GACAGGTATACATGGAAAATTTACTCGGAGAGGCTGATGACTTTGGCTGGTGTATATGGTTTCTGGAAGC

TTGTTTCAAAACTCGAGAGGCGTGAAACTAGACGATACCTCGAGATGTTCTACATTCTCAAGTTCCGTGA

GTTGGTAAAATCTGTTCCTCTAGCAGTTGATGAGAAGCAGTGAGGATTTTGCAAAGGAATAAATAACGTG

GAGCATACCACTCTATCTATGATAATTTTTTTTTTGTTCTTCTTGTTTGTTGTGTAGTTAAACTAATTAA

GTGCACTGAGTTTCTAATAGATCTCAAATAGCTAAACGTTACTATCTCCTATTTTAATAGCGCTATACTT

TAATTTGTCAAATA

Highlighted in yellow is the sequence used for creating the SUS4-RNAi construct
