# Supplementary figures and images for: Suppression of sucrose synthase affects auxin signaling and leaf morphology in tomato
Source: PLoS One. 2017 Aug 7;12(8):e0182334. doi: 10.1371/journal.pone.0182334 (PMC5546705; doi:10.1371/journal.pone.0182334)

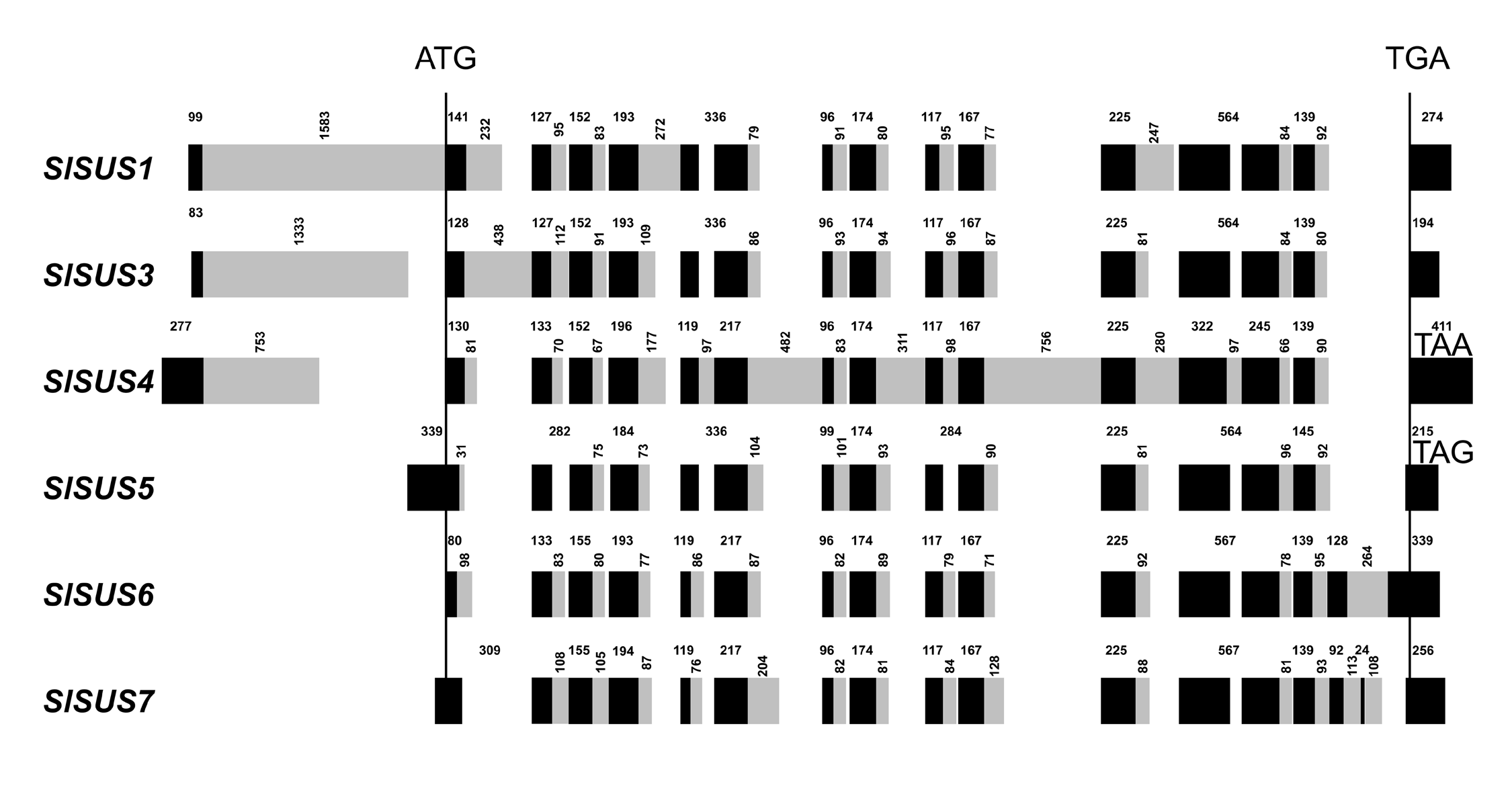

Supplement: S1 Fig — Comparative schematic presentation of genomic SlSUS sequences: SlSUS5, SlSUS6 and SlSUS7 genomic and cDNA sequences were obtained from the Sol Genomics Network (https://www.solgenomics.net/) and aligned with the SlSUS1,3&4 gene structure described by Goren et al. [10]. Exons (black) have nearly the identical size in all isoforms, with the introns (gray) identically placed, but more variable in size. Numbers denote the size (bp) of exons (horizontal) and introns (vertical). ATG, start codon; TGA, TAA, TAG, stop codons. (TIF) [file pone.0182334.s004.tif]

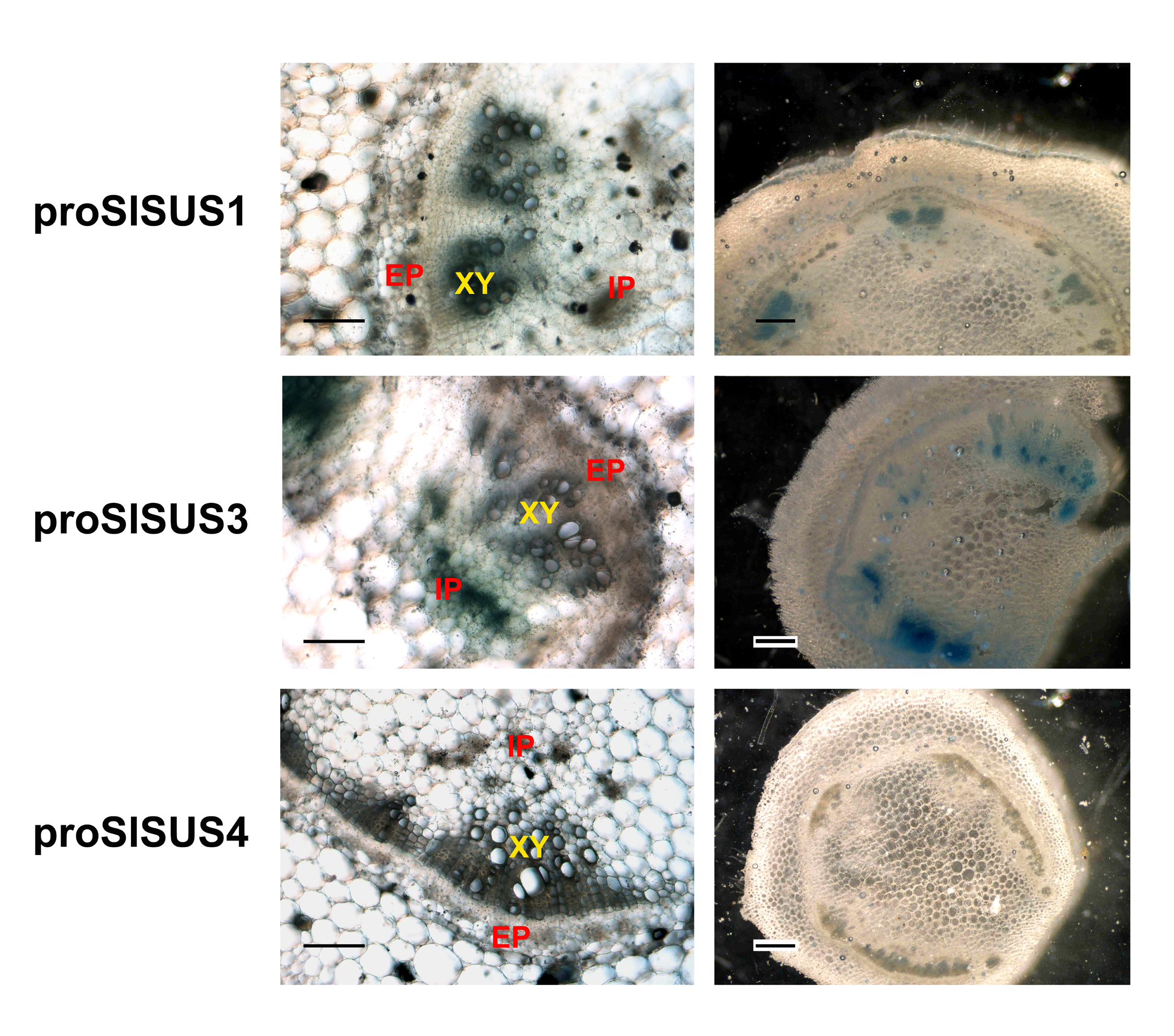

Supplement: S2 Fig — Free-hand cross-sections of GUS stained petioles observed under microscope (left column, Bars– 200 μm) or under dissecting microscope (right column, Bars– 500 μm); EP–external phloem; IP–internal phloem; XY–xylem vessel members. (TIF) [file pone.0182334.s005.tif]

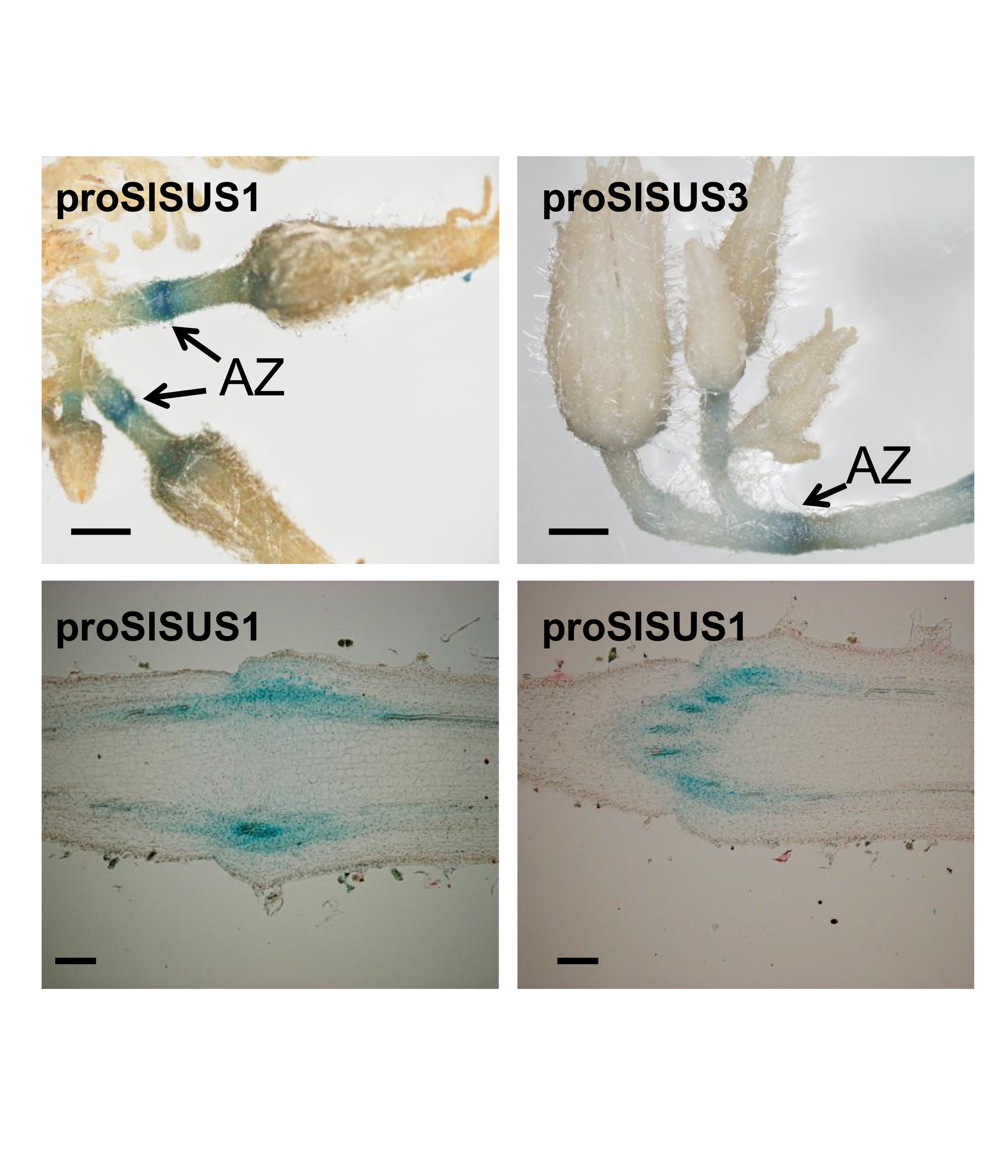

Supplement: S3 Fig — (A) proSlSUS1 plants exhibit GUS staining in the inflorescence abscission zones. (B) proSlSUS3 plants exhibit GUS staining in the inflorescence abscission zones. (C, D) Longitudinal cross-sections of proSlSUS1 inflorescences show that GUS staining is primarily seen around the vascular tissues. (A, B) Bar– 1 mm; (C, D) bar– 100 μm. (TIF) [file pone.0182334.s006.tif]

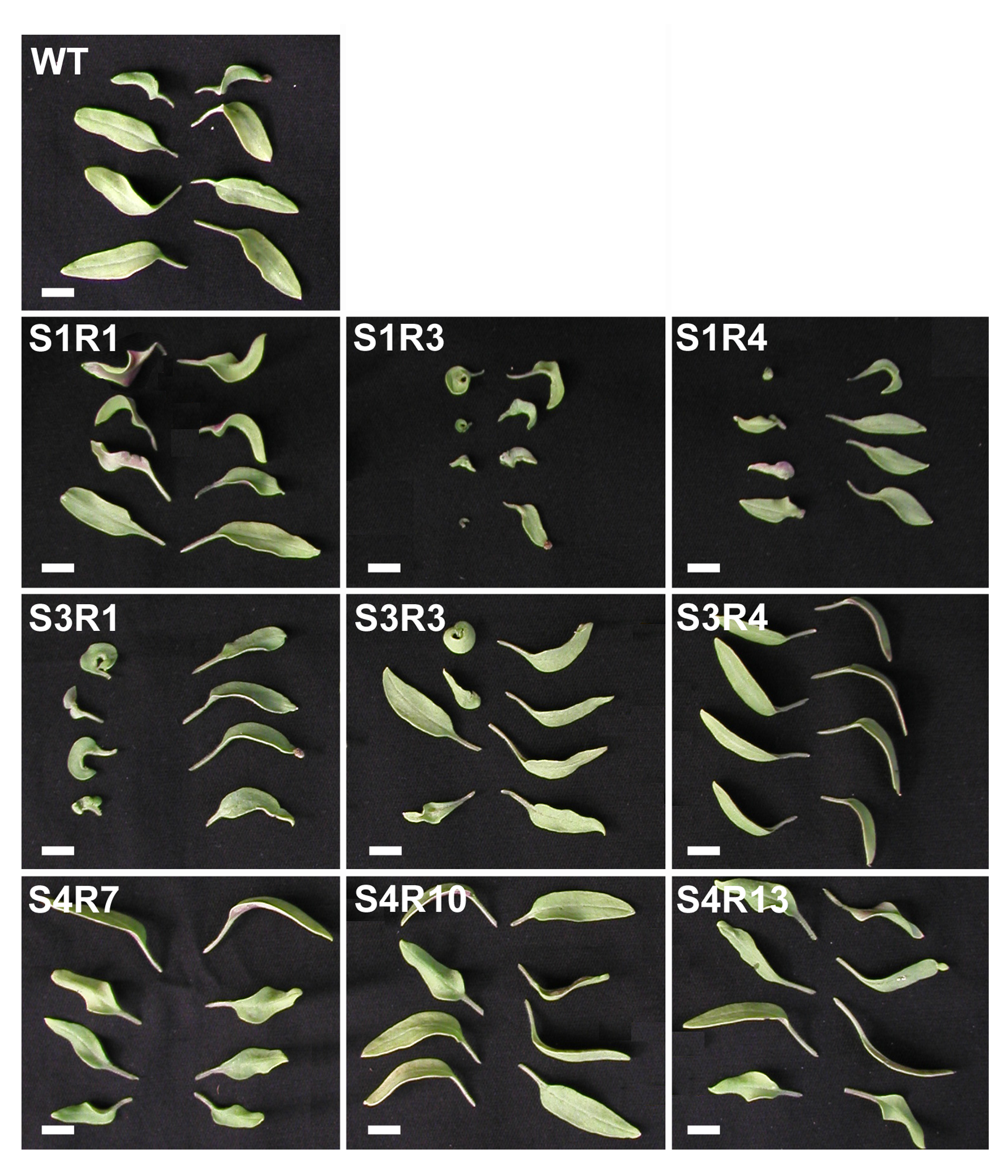

Supplement: S4 Fig — Each pair of cotyledons was taken from a single seedling. Bar– 1 cm. (TIF) [file pone.0182334.s007.tif]

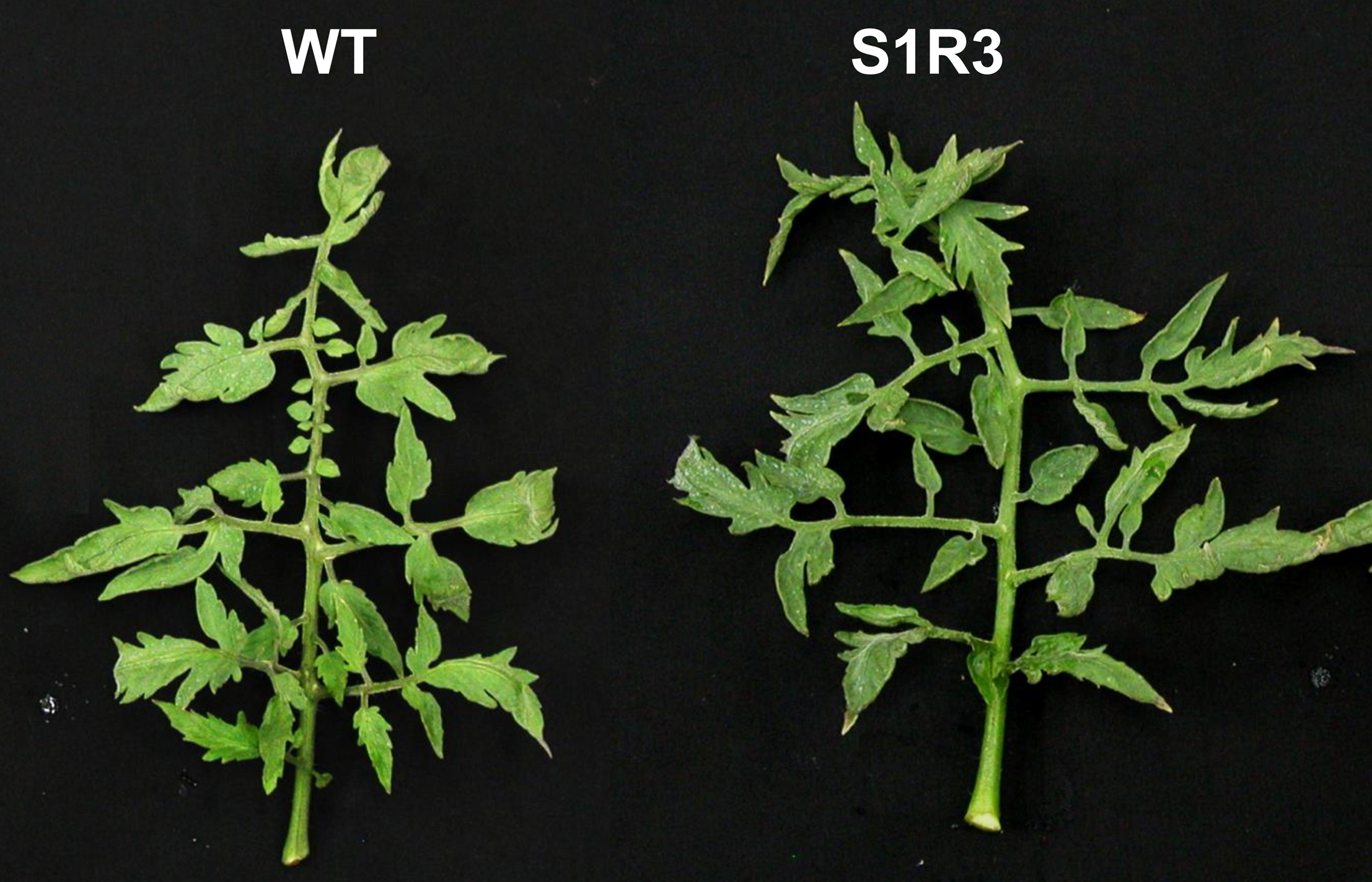

Supplement: S5 Fig — Mature leaves of wild-type (WT) and S1R3 line. (TIF) [file pone.0182334.s008.tif]

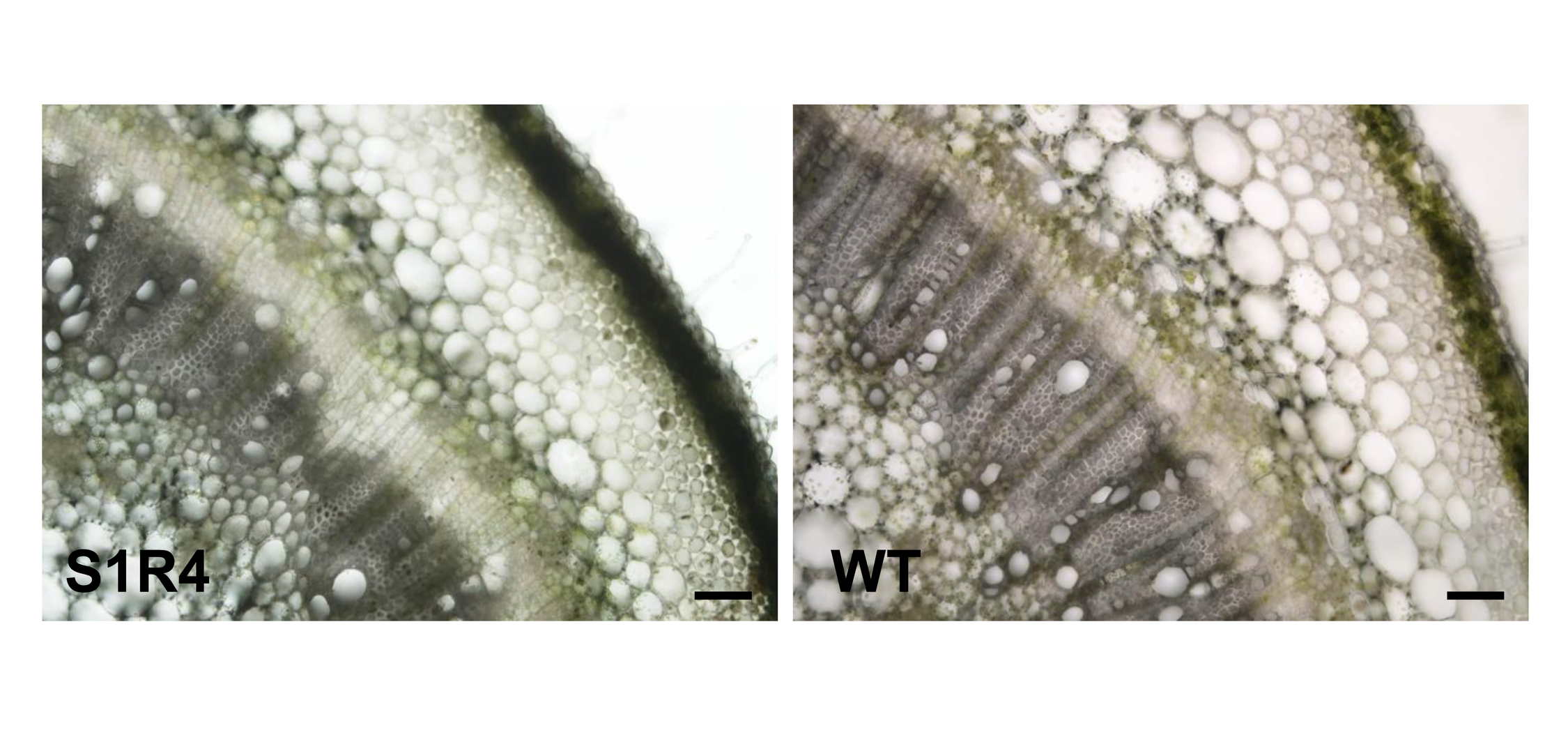

Supplement: S6 Fig — Light microscopy of free-hand cross-sections of S1R4 (A) and WT (B) petioles from mature leaves. Bar– 0.5 mm. (TIF) [file pone.0182334.s009.tif]
